# The role of agri-environment schemes in conservation and environmental management

**DOI:** 10.1111/cobi.12536

**Published:** 2015-05-21

**Authors:** Péter Batáry, Lynn V Dicks, David Kleijn, William J Sutherland

**Affiliations:** * Agroecology, Georg-August-UniversityGrisebachstr. 6, D-37077, Göttingen, Germany; † Conservation Science Group, University of Cambridge, Department of ZoologyDowning Street, Cambridge, CB2 3EJ, United Kingdom; ‡ Resource Ecology Group, Wageningen UniversityDroevendaalsesteeg 3a, 6708 PB, Wageningen, The Netherlands; § Alterra, Animal Ecology TeamP.O. Box 47, 6700 AA, Wageningen, The Netherlands

**Keywords:** agricultural intensification, Common Agricultural Policy, Europe, European Union, farmland, field margin, grassland, organic management, Europa, intensificación agrícola, margen del campo, manejo orgánico, pastizal, Política Agrícola Común, tierra de cultivo, Unión Europea

## Abstract

Over half of the European landscape is under agricultural management and has been for millennia. Many species and ecosystems of conservation concern in Europe depend on agricultural management and are showing ongoing declines. Agri-environment schemes (AES) are designed partly to address this. They are a major source of nature conservation funding within the European Union (EU) and the highest conservation expenditure in Europe. We reviewed the structure of current AES across Europe. Since a 2003 review questioned the overall effectiveness of AES for biodiversity, there has been a plethora of case studies and meta-analyses examining their effectiveness. Most syntheses demonstrate general increases in farmland biodiversity in response to AES, with the size of the effect depending on the structure and management of the surrounding landscape. This is important in the light of successive EU enlargement and ongoing reforms of AES. We examined the change in effect size over time by merging the data sets of 3 recent meta-analyses and found that schemes implemented after revision of the EU's agri-environmental programs in 2007 were not more effective than schemes implemented before revision. Furthermore, schemes aimed at areas out of production (such as field margins and hedgerows) are more effective at enhancing species richness than those aimed at productive areas (such as arable crops or grasslands). Outstanding research questions include whether AES enhance ecosystem services, whether they are more effective in agriculturally marginal areas than in intensively farmed areas, whether they are more or less cost-effective for farmland biodiversity than protected areas, and how much their effectiveness is influenced by farmer training and advice? The general lesson from the European experience is that AES can be effective for conserving wildlife on farmland, but they are expensive and need to be carefully designed and targeted.

El Papel de los Esquemas Agro-Ambientales en la Conservación y el Manejo Ambiental Batáry et al.

**Resumen:**

Más de la mitad de las tierras europeas está bajo manejo agrícola y así ha sido durante milenios. Muchas especies y ecosistemas de interés de conservación en Europa dependen del manejo agrícola y están mostrando una declinación continua. Los esquemas agro-ambientales (EAA) están diseñados en parte para encarar esto. Los esquemas son una gran fuente de financiamiento para la conservación dentro de la Unión Europea (UE) y el mayor gasto de conservación en Europa. Revisamos la estructura de los EAA actuales a lo largo del continente. Desde que en 2003 una revisión cuestionó la efectividad general de los EAA para la biodiversidad, ha habido una plétora de estudios de caso y meta-análisis que examinan su efectividad. La mayoría de las síntesis demuestran un incremento general en la biodiversidad de las tierras de cultivo en respuesta a los EAA, con la magnitud del efecto dependiente de la estructura y el manejo del terreno circundante. Esto es importante a la luz del crecimiento sucesivo de la UE y las continuas reformas a los EAA. Examinamos el cambio en la magnitud del efecto a través del tiempo al fusionar los conjuntos de datos de tres meta-análisis recientes y encontramos que los esquemas implementados después de la revisión de los programas agro-ambientales de la UE en 2007 no fueron más efectivos que los esquemas implementados antes de la revisión. Además, los esquemas enfocados en las áreas fuera de producción (como los márgenes de campo y los setos vivos) son más efectivos en el mejoramiento de la riqueza de especies que aquellos enfocados en las áreas productivas (como los cultivos arables y los pastizales). Las preguntas sobresalientes de la investigación incluyen si los EAA mejoran los servicios ambientales, si son más efectivos en las áreas agrícolas marginales que en las áreas de cultivo intensivo, si son más o menos rentables para la biodiversidad de las tierras de cultivo que las áreas protegidas, y en cuánto influye sobre su efectividad los consejos y el entrenamiento dado a los granjeros. La lección general de la experiencia europea es que los EAA pueden ser efectivos para la conservación de la vida silvestre en las tierras de cultivo, pero son caros y necesitan ser diseñados y enfocados cuidadosamente.

## Introduction

There is an obsession with farmland conservation in Europe that is not understood in other parts of the world (Stoate et al. 2009). Visiting conservationists are often amazed to discover that European national parks are grazed by livestock or actively cultivated and that the small remaining area of woodland may be cut for the sake of conservation. The core explanation is the long history of intensive human management. Europe has been occupied by humans for at least 700,000 years (Parfitt et al. 2005), while the domestication of crops in the Fertile Crescent of southwestern Asia about 10,000 years ago led to a rapid spread of agriculture across Europe and radical social and ecological change. Much of the recent European landscape was established by Roman times. As Rackham (1986) states “… England in 1945 would have been instantly recognizable by Sir Thomas More [1478–1535], and some areas would have been recognized by the Emperor Claudius [in AD 43].”

For thousands of years, European lowlands have been grazed and cultivated, wetlands cut for reed or sedge, and uplands grazed by livestock, while woodlands are largely coppiced (cut regularly at the base to provide poles) or pollarded (cut above grazing height to provide poles) and interspersed with large trees maintained as standards (felled when mature to provide large beams). As a result, over large areas there is little natural vegetation. Much of the European countryside is an artificial landscape, where areas are kept open not by natural disturbance and indigenous herbivores but by farming and farm animals.

This artificial landscape is loved by human residents and visitors from abroad. Many highly valued species require disturbance, and leaving habitats unmanaged to allow natural succession often results in dramatic loss of these species (Thomas 1991). As a result, many protected areas in Europe are managed in ways that reflect traditional agricultural practices. This represents an interesting cultural conflict. Although agricultural intensification is generally considered the most important driver of global terrestrial biodiversity loss, through habitat loss, habitat fragmentation, and habitat conversion (Foley et al. 2011), in Europe agriculture itself has long been understood as part of the solution. Much of current European nature conservation aims to halt the on-going loss of farmland biodiversity, evolved during millennia of extensive management (Sutherland 2004), and abandonment of agriculture is generally seen as a threat to biodiversity (Queiroz et al. 2014). In this sense, Europe is different from other continents, particularly the Americas, where areas of high biodiversity interest are rarely in use for commercial production of food, and agricultural practices are not prominent in conservation strategies (Boitani & Sutherland 2015 [this issue]).

Since the early 20th century, both the mass production of nitrogen fertilizers and the development of pesticides have greatly increased agricultural yields (Smil 1999). The increasing use of agrochemicals was accompanied by widespread mechanization, especially after the Second World War. This resulted in intensification at field scale as well as at larger scales (Batáry et al. 2011). The trajectories of change varied among countries, which differed in their political ideologies and biogeographies. In northwestern Europe, considerable areas of species-rich semi-natural grassland and heath were effectively destroyed by plowing, chemical application, and re-sowing (either with crops, grasses or, in some cases, commercial forest) during the 20th century (e.g., Fuller 1987). Since then, ongoing drivers of biodiversity loss have included the shift to autumn sown cereals, improved efficiency of pesticides, and specialization of farm systems, which has led to a loss of mixed farming and hedgerow removal to create larger fields, especially in arable areas (Robinson & Sutherland 2002). In the central and eastern countries of the Eastern Bloc, collectivization of farms resulted in large co-operatives, where field roads, hedgerows, and field margins were eliminated to merge small fields into large-scale agricultural systems (e.g., Báldi & Batáry 2011; Sutcliffe et al. 2015). In southern European countries around the Mediterranean, 20th century agricultural land-use change was characterized by abandonment of farmland, natural and artificial reforestation associated with declining rural population densities (e.g., Debussche et al. 1999; Padilla et al. 2010), and intensification of agriculture in accessible plains (as in central Spain). In all these contrasting contexts, agri-environment schemes (AES) are one of the main practical 21st century solutions to mitigate or reverse the consequent biodiversity loss because they directly support the necessary agricultural management.

For this paper, we reviewed the history, current use, and effectiveness of AES as a conservation tool in Europe. We considered the conceptual framework that has been developed to interpret the ecological findings and the implications of research on the human factors that influence farmer uptake or acceptance of the schemes. We conducted 2 new meta-analyses to determine whether AES are becoming more effective over time and whether changing management in productive or non-productive areas benefits biodiversity. We also identified outstanding policy-relevant research questions that cannot currently be answered using formal meta-analysis, due to data deficiency. Finally, we considered what can be learned about the use and cost-effectiveness of AES from the European experience.

## A Short History of Agri-Environment Schemes in Europe

**Figure 1:**
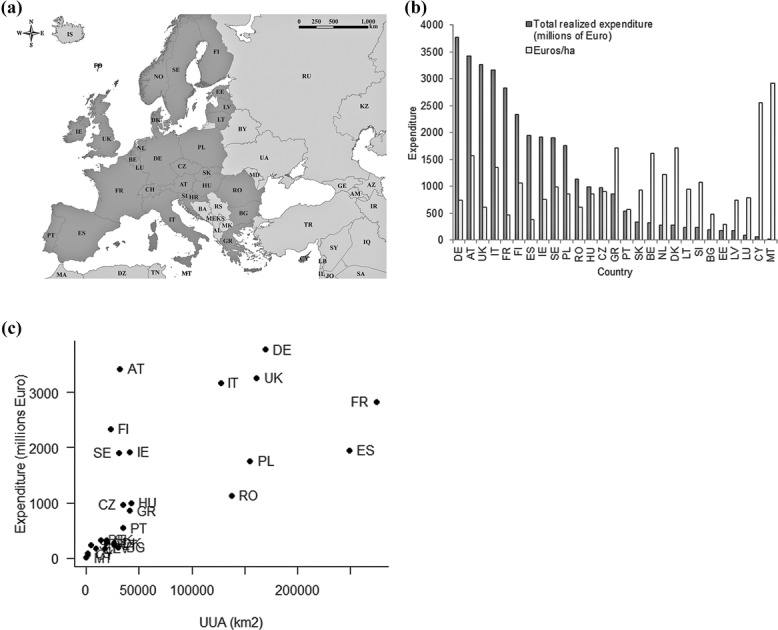
(a) Countries (codes defined in Supporting Information) in Europe where agri-environment schemes (AES) exist (dark gray). (b) Total realized expenditure spent on AES in 2007–2013 (dark gray) and total realized expenditure spent on AES in 2007–2013 per area under AES (light gray) (no data available for Croatia, Norway, and Switzerland). (c) Utilized agricultural area (UAA) relative to total realized expenditure on AES in 2007–2013. Data for (b) and (c) derived from European Network for Rural Development (2014).

Although some northwestern European countries had agri-environment programs predating any European regulations, most European AES can be traced back to the Agricultural Structures Regulation of 1985 (European Union [EU] Regulation 797/85). They were conceived as a mechanism to compensate farmers for loss of income associated with appropriate, less intensive management of environmentally sensitive areas in response to the changes described above and largely driven by a few countries of the north and west (Hodge et al. 2015 [this issue]). In 1987 an amendment (EU Regulation 1760/87) allowed up to 50% of the cost of environmentally sensitive areas to flow from the Common Agricultural Policy (CAP), and in 1992 AES became compulsory for all EU Member States (EU Regulation 2078/92). They are one aspect of the Rural Development pillar of CAP. Each Member State designs its own schemes. Currently, a diversity of AES exists in the 28 Member States of the EU and in Switzerland and Norway, which are not Member States (Fig. 1a; Supporting Information). We confined our synthesis to 30 countries rather than the entire continent.

Because they provide income for conservation, AES have become the main tool to conserve biodiversity on European farmland and are often used to fund management in protected areas or designated sites. Within the EU, AES have always been, and remain, voluntary for land managers, although in the latest reform of CAP in 2014 certain management practices designed as AES became obligatory for farmers to qualify for their basic subsidy (Pe'er et al. 2014).

AES are important for conserving farmland areas designated by EU countries, Switzerland, and Norway as of “high nature value” (Lomba et al. 2014) in that they preserve genetic diversity of livestock, protect a diversity of agro-ecosystems types, and produce food with a lower environmental and ecological footprint. Many schemes have clear objectives to reduce water pollution, enhance access to the countryside and protect cultural landscapes and heritage, as well as protecting biodiversity. Almost all countries have AES that support organic farmers, based on an underlying assumption that organic farming is good for the environment (Tuck et al. 2014).

The role of AES schemes has shifted over time. Their initial purpose was to protect threatened habitats or landscapes. Over time, the emphasis changed to prevention of species’ loss, especially farmland birds, across agricultural land. More recently, emphasis is shifting to the application of AES to improve and maintain ecosystem services, such as pollination and biocontrol (Ekroos et al. 2014).

Schemes can be classified as horizontal or zonal (i.e., targeted) (Kleijn & Sutherland 2003). Horizontal schemes usually combine environmental protection with nature conservation objectives and can be applied throughout a country. They are designed to fit easily into farm management systems; they are not too demanding or directly support management farmers are doing anyway, such as organic management. Zonal schemes target areas with high nature value. They generally require bespoke management for target species or ecosystems, and farmers are often obliged to seek expert advice in developing management plans.

## Big Spending for Conservation

Budgets for AES are substantial and for most countries usually equal or exceed the amounts of money spent on wildlife conservation through other routes. For example, in 2005 the Dutch budget for conservation in protected areas was €48.8 million, while that for AES with biodiversity objectives was €42.1 million (MNP 2007). In England, total expenditure on AES, including measures with non-biodiversity objectives, was €375 million/year from 2007 to 2013 (European Network for Rural Development 2014). The total annual expenditure of the government's nature conservation agency for England was much lower, around €250 million in 2013–2014 (Natural England 2014). In new EU member states this difference can be larger. For example, in 2008 the Hungarian budget for nature conservation was roughly €41.0 million (Hungarian Government 2009), while total expenditure on AES was €117.6 million (Hungarian Ministry of Agriculture and Rural Development 2009). The European Commission spent €3.23 billion on AES in 2012, a figure two orders of magnitude higher than the cost of managing Natura 2000 sites (Maiorano et al. 2015 [this issue]) that year, which was €39.6 million (Pe'er et al. 2014).

The total amount of public expenditure on AES in each EU Member State for 2007–2013, including co-financing at national levels, is strongly correlated with the amount of agricultural land in each country (Fig. 1c) (Spearman rank rho = 0.83, *P* < 0.001), although some countries are relative outliers. Spain and France spend less than would be expected from their agricultural area, while Austria spends more. The proportion of agricultural land under the schemes varies greatly across countries, from 6% in Denmark to 95% in Finland (Supporting Information). This means the intensity of spending also differs among countries, as illustrated by the amount of money spent per hectare of AES area (Fig. 1b); there is a tendency for more focused spending in smaller countries.

Future spending on AES is very likely to be lower in all countries, following reforms of CAP enacted at the end of 2013 (Pe'er et al. 2014). The budget for Rural Development Programmes, of which AES are part, will be 18% less by 2020. Moreover Member States have been given the choice to shift funds out of Rural Development to directly support farmers. In the coming years, differences among countries in AES spending will therefore increase.

## Ecological Effectiveness of European Agri-Environment Schemes

Given the huge expenditure on European AES, it is important to ask whether they improve biodiversity outcomes. The first well-designed studies examining the ecological effects of AES were published in the early 2000s. Kleijn and Sutherland (2003) reviewed published peer-reviewed and gray literature on the effectiveness of AES with biodiversity targets and concluded that about half of the schemes lack positive effects on biodiversity. Successful schemes focus mainly on specific (rare) species and are often supervised by scientists or volunteers. Non-targeted schemes to enhance biodiversity usually benefit common species or have no overall impact.

Since that review there has been a wealth of published papers on the subject and a number of important Europe-wide reviews (Supporting Information). These demonstrate that AES generally enhance biodiversity locally, usually with modest increases in species richness or abundance of common species. Studies have been mainly of intensively farmed areas; little work has been done on effectiveness of schemes in areas with more extensive agriculture (Kampmann et al. 2012).

Based on these studies a theoretical framework has been developed. The effectiveness of AES at attracting wild species is influenced by landscape structure, land-use intensity, and the ecological contrast created by AES (Kleijn et al. 2011). The hypotheses on the relationship between effectiveness and landscape structure and between effectiveness and ecological contrast have both been confirmed (Batáry et al. 2011; Scheper et al. 2013; Hammers et al. 2015). In their meta-analysis, Batáry et al. (2011) found that in cropland areas AES are effective in simplified but not in complex landscapes. This was further confirmed in a meta-analysis on pollinators (Scheper et al. 2013) and by Tuck et al. (2014), who showed that the positive effects of organic farming on biodiversity increased as the amount of cropland increased. However, the suggested relationship between effectiveness and land-use intensity has not been confirmed, possibly because most research has been done in countries dominated by intensive farming, such as the United Kingdom and Germany (Dicks et al. 2013*a*), and has not specifically incorporated an intensification gradient. There is almost no evidence yet on whether this attraction of wild species to AES land represents a stabilization and increase of plant and animal populations or a local concentration of these populations with concurrent dilution in other nearby areas (but see Morandin & Kremen 2013).

We addressed 2 specific issues by merging the data sets of 3 recent meta-analyses on the effects of AES on species richness (Batáry et al. 2011; Scheper et al. 2013; Tuck et al. 2014). We imposed the following restrictions: only studies from the 28 European Member States, Norway, and Switzerland were included; studies were excluded if the number of replicates was fewer than three experimental or control areas; studies performed at plot level (i.e., within-field experiments) were excluded. This resulted in a data set with 284 observations from 103 studies (the entire data set is in Supporting Information).

We used the unbiased standardized mean difference (Hedges’ *g*) as a common effect size in our analyses, originating from the above meta-analyses. Effect size was positive if species richness was higher in the AES than in the control fields. For the error estimate, we used the non-parametric variance estimates of each effect size, which is based on few assumptions and may be less constrained by the assumptions of large sample theory (Hedges & Olkin 1985). We carried out statistical analyses in the metafor package (Viechtbauer 2010) of R (R Development Core Team 2013). Funnel plots, regressions test for funnel plot asymmetry, and calculated fail-safe numbers all showed no sign of publication bias, either in the entire data set or in the 2 meta-analyses presented (Supporting Information). However, our meta-analyses shared with the three previous meta-analyses a strong geographic bias of study areas towards Northern and Western Europe. This issue was previously highlighted by Tryjanowski et al. (2011) and recently by Sutcliffe et al. (2015). They concluded that new eastern EU Member States had adopted Western European type AES designed for intensively farmed landscapes. In the extensively farmed areas in the new member states such AES seem to be ineffective or even have negative effects on biodiversity. Therefore, there is a great need for better locally adapted AES.

### Effectiveness of Agri-Environment Schemes over Time

The regular reforms of CAP allow countries to use novel scientific insights and modify their agri-environmental programs to increase their efficiency. As a result national agri-environmental programs change substantially every 7 years. Dicks et al. (2013*b*) questioned whether scientific evidence was used to improve policy efficiency during the most recent CAP reform. After 25 years of AES in Europe and almost 15 years of high-quality research on their effectiveness, it is possible to ask whether the effectiveness of the schemes has improved as policy experience and scientific evidence accrued over time.

If evidence was being taken into account, findings from studies in the early 2000s, which mostly covered AES implemented in the 2000–2006 budget period or before, would be reflected in the designs of schemes in the 2007–2013 budget periods. This may be expected to result in increased effectiveness in the second budget period. To test this, we used a mixed-effects meta-regression model in which budget period was the moderator variable (Supporting Information).

**Figure 2:**
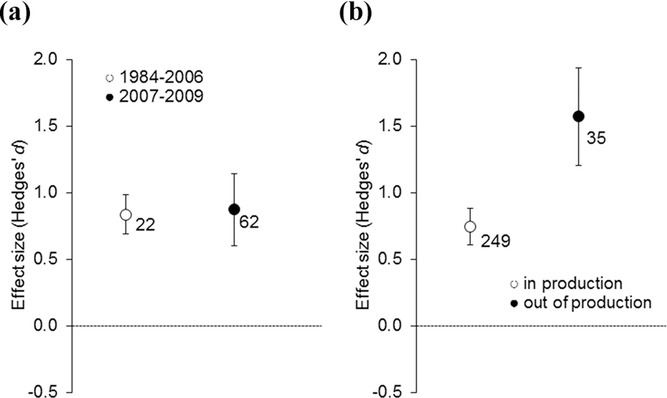
(a) Changes in effectiveness of agri-environment schemes over time as shown in studies published from 1984 to 2006 compared with studies published from 2007 to 2009 and (b) differences in species diversity between control areas and areas in production (such as fields under organic management) and areas out of production (such as field margins and hedgerows). Shown are mean effect sizes and 95% CI. The mean effect size is significantly different from zero if the CIs do not overlap with zero. Numbers near symbols indicate sample size.

We found that schemes implemented after 2007 were not more effective than schemes implemented before 2007 (Fig. 2a, Supporting Information). Although AES were effective in both periods, there was no sign of improvement in effectiveness over time.

Of course, we cannot conclude directly from this that science is not being used to improve design of the schemes. There are other possible explanations for the lack of improvement over time. We know that biodiversity is still degrading and agricultural landscapes are still changing in Europe, and both of these could potentially decrease the effectiveness of AES as a result of the reduced pool of species available to colonize and benefit from the scheme. Alternatively, there might be a time-delay effect, meaning that the positive effect of research on AES will appear farther in the future (Weis 2001).

It is unfortunate that there is no evidence yet of AES becoming more effective over time, as such a change might have compensated to some extent for forthcoming reductions in AES budgets (Pe'er et al. 2014). Policy makers might argue that elements of AES, such as field margins left out of production, become obligatory across Europe as “compulsory greening measures” under the direct payments pillar of CAP from 2014–2020 and that this would compensate for loss of AES coverage. However, recent analyses of the compulsory greening measures show that effective elements of AES have generally not been incorporated (Dicks et al. 2013*b*; Pe'er et al. 2014). Rather than being obligatory, the greening measures that are similar to AES (known as ecological focus areas) apply to just over half the farmed area of Europe, due to the exemption of farms of <15 ha of arable land (Pe'er et al. 2014).

### Effectiveness of Agri-Environment Schemes in Productive versus Non-Productive Areas

AES can be classified according to whether they apply to non-productive areas, such as field boundaries and wildflower strips (sometimes called off-field practices [Garibaldi et al. 2014]), or productive areas, such as arable crops or grasslands (sometimes called on-field practices). Schemes targeting non-productive areas include hedgerows, sown or naturally regenerated field margins, or simply taking areas of land out of production for different conservation purposes. We call these out-of-production schemes (Supporting Information). In contrast, in-production schemes support environmentally sensitive approaches to the management of land that is used to grow crops or feed livestock. For example, the use of agrochemicals might be reduced or prohibited or certain management actions, such as mowing grassland, might be restricted. The most widespread in-production scheme is organic farming.

In our second meta-analysis, we used a mixed-effects meta-regression model with management type as a moderator variable. We found that out-of-production schemes were much more effective at enhancing species richness than in-production schemes (Fig. 2b, Supporting Information). A possible explanation may be that most of the out-of-production schemes we examined evaluated measures that take agricultural land out of production, such as the establishment of wild-flower strips. The conversion of crop monocultures to semi-natural habitat results in a much larger increase in resource availability (i.e., creates a larger ecological contrast) for a wider range of species than measures such as organic farming, reducing stocking rates, or restricting fertilizer application rates that are typical for in-production schemes. Schemes promoting the establishment of wildflower strips may also be better targeted to the conservation of a given species group than in-production schemes because they often specifically address a resource that is limiting population growth or size (e.g., floral resources for flower visiting insects). Many in-production schemes do not address specific species groups; rather, they aim to enhance biodiversity in general as one of several targets, alongside improvements in other ecosystem characteristics or services.

Targeting the needs and spatial distribution of specific species groups is most likely more important than whether schemes prescribe measures on or off land that is being used for farming. Targeted schemes tend to be more effective than untargeted schemes (Kleijn & Sutherland 2003; Wilson et al. 2009), and better spatial targeting of in-production schemes can greatly benefit rare and declining species (Pywell et al. 2012). In many countries, there is a move toward better targeting of AES, either toward particular declining species groups or landscapes where they are likely to be effective. As this is being incorporated into AES and implemented between now and 2020, one might expect a review similar to this one in 2025 to be able to show an increase in effectiveness of AES over time.

It is important to appreciate that species richness is just one measure of diversity, although this is the one most easily understood and used by policy makers. We think that the importance of this measure is overrated and other variables characterizing biodiversity should be applied in primary studies and analyzed (if sufficient studies are available) in meta-analyses (e.g., the meta-analysis on functional diversity by Flynn et al. [2009]). An additional fundamental point is that in-production and out-of-production options typically support different communities. In-production options select for species adapted to the highly disturbed, cropped areas of fields, for example, in contrast to out-of-production options (see the example of arable weeds in Storkey et al. [2012]).

## The Human Factor

In addition to research on the ecological effectiveness of AES, there is a body of work on how to ensure that AES are palatable to farmers and therefore effective at changing farmer behavior. This is important because AES are always voluntary (but see recent CAP reform [Pe'er et al. 2014]). Uptake of specific AES options is a key element of their success and does not always correlate with ecological effectiveness. For example, Hodge and Reader (2010) found that the vast majority of options taken up in the first 5 years of entry level stewardship (a horizontal scheme) in England were the straightforward field corner and grass margin options that require little change of management or resource investment. Evaluation of synthesized evidence shows that these are not the most effective AES options for enhancing biodiversity (Dicks et al. 2013*b*).

Studies on motivations of farmers to take up AES or environmental management have repeatedly demonstrated that farmer attitudes are important in explaining uptake of environmental measures (e.g., Defrancesco et al. 2008; Sattler & Nagel 2010). As well as the effect of general attitude, scheme adoption is linked to utilitarian motivations, such as payment rate and ease of fit within existing farm practice (e.g., Defrancesco et al. 2008; Sutherland 2010). Many authors have pointed out that AES intended to support biodiversity should be designed with farmer circumstances and attitudes in mind (e.g., Herzon & Mikk 2007; de Snoo et al. 2013), indicating a need for ecologists and social scientists to work together. Herzon and Mikk (2007) found that views of biodiversity among Finnish and Estonian farmers were largely restricted to the realm of wild nature outside the farmed environment. This implies a need to demonstrate to farmers when they can directly benefit from measures to promote functional ecological groups of biodiversity, such as pollinators, natural enemies, or soil biodiversity.

## Future Research

### Effectiveness of AES at Enhancing Ecosystem Services

The value of ecosystem services to agriculture has been much discussed recently (e.g., Power 2010; Kremen & Miles 2012). For some services, such as food production, pest regulation, pollination, and soil nutrient cycling, farmers themselves are direct beneficiaries because their yields and input requirements are directly affected. Other services, such as air and water quality or enjoyment of cultural landscapes, are public goods (i.e., the main beneficiaries are outside the farm business). The role AES can and should play in maintaining ecosystem services is still under discussion. There is a clear mandate for CAP to support delivery of public goods from agriculture (European Commission 2010) but not to support actions that directly increase farm income.

The effectiveness of specific AES options at delivering ecosystem service benefits has only just started to be tested. For example, a small number of studies outside Europe have demonstrated benefits to crop pollination from wildflower strips or patches (Garibaldi et al. 2014), and there is some evidence that vegetated buffer strips can enhance water quality (Zhang et al. 2010). The combined effects of specific AES options on multiple ecosystem services are still poorly understood.

### Effectiveness of AES in Agriculturally Marginal Areas versus Intensively Farmed Areas

In Europe agriculturally marginal areas, where the productivity of land is limited by biophysical or socio-economic constraints, are currently home to the highest concentrations of biodiversity and host the largest populations of threatened species (Tryjanowski et al. 2011). Many of them typically occur in new central and eastern Member States (Sutcliffe et al. 2015). These areas are under pressure from agricultural intensification and abandonment. Counteracting farmland abandonment in marginal areas is an important objective of AES in many countries, yet surprisingly few studies have examined the effects of AES on marginal farmland. What limited evidence there is suggests that AES can be very effective on low-intensity farmland. Schemes effectively support threatened birds in low-input cereal steppes in Central Spain (Kleijn et al. 2006), bird richness in environmentally sensitive areas in Hungary (Kovács-Hostyánszki & Báldi 2012), and species-rich plant communities in the Swiss Alps (Kampmann et al. 2012). Weis (2001) conducted an illustrative study in the German Eiffel mountain range, where many low-productive species-rich grasslands had been abandoned or afforested since the late 1960s, but then AES were introduced in 1986 that paid farmers to reintroduce sheep grazing on abandoned grasslands. Weis (2001) compared trends in plant species richness in plots where grazing had recommenced and plots where sheep were kept out. In 1999 species richness in grazed plots had increased by 20%, while species richness in ungrazed plots had decreased by 17%. The population size of a range of threatened orchid species increased by 50–500% in grazed plots. However, it took 8–10 years before the first positive effects became apparent, which may explain why this has been an unpopular research topic. Previous AES were designed solely to maintain biodiversity (e.g., by reintroducing extensive management) and not to restore it completely (Kleijn et al. 2009), so it was cheaper to execute these schemes in marginal areas than in intensive areas. More studies are needed, however, before general conclusions can be drawn about the effectiveness of AES in agriculturally marginal areas.

### Cost-effectiveness of Agri-Environment Schemes Compared with other Conservation Approaches

As a conservation strategy, AES focus on reducing the impact of agricultural activities on species that inhabit the agricultural landscape. They are not the only possible route to protect such species. Another major conservation tool is protected areas, which can also be applied in agricultural landscapes. In some countries, there are protected sites managed as working farms for farmland wildlife (e.g., Moyse 2013). Little is known about the relative efficiency of these different strategies to protect farmland biodiversity.

A notable exception is the case of meadow bird conservation in the Netherlands. In 2008 €21 million was spent on AES targeting meadow bird conservation on large areas of farmland. In the same year, meadow bird conservation in the spatially much more restricted protected areas cost €4 million (van Paassen & Teunissen 2010). Settlement densities are much higher in protected areas than on farmland with meadow bird schemes, resulting, at the national level, in slightly more meadow birds breeding in protected areas than on farmland with meadow bird schemes (PBL 2009). Furthermore, on average, meadow birds show positive trends in protected areas but negative trends on farmland with meadow bird schemes (van Egmond & de Koeijer 2006). This suggests that, for this particular species group, protected areas are much more efficient than AES. However, it might be that most protected areas in the Netherlands are too small to maintain viable meadow bird populations in the long run, especially when they are bordered by inhospitable high-intensity grasslands or built-up areas that are generally avoided by these ground-nesting birds. So the apparent higher cost-effectiveness might be an illusion, hiding an extinction debt.

The comparison in cost-effectiveness between AES and protected areas is important because both are funded with public budgets and both impact the potential for food production. Investing in one strategy does not necessarily mean there is less money available for the other strategy because the source of funds for AES has a very different underlying purpose – to support farm incomes and generate public goods from agriculture. Even so, cost-effective conservation is of interest to policy makers (further discussion in Supporting Information).

### Importance of Training and Advice to the Effectiveness of Agri-Environment Schemes

There has been little research on the link between farmer training or advice and the effectiveness of AES. Farmers are trained in agricultural production and have seldom experienced specific training or education in environmental management. Yet managing land for environmental outcomes requires a different set of skills and knowledge. Zonal AES schemes usually incorporate an element of training or advice. In the United Kingdom, zonal schemes are much more beneficial to bird diversity per unit cost than simplified horizontal schemes, despite the fact that a much larger proportion of the funding goes into setting up and checking the implementation rather than directly to farmers (Armsworth et al. 2012).

Horizontal AES often do not incorporate farmer training or advice (but see Marja et al. 2014), and this could be a reason for their relatively low effectiveness. One research project in the United Kingdom demonstrated that training farmers increases their confidence and develops a more professional attitude to agri-environmental management (Lobley et al. 2013). The same project also demonstrated ecological benefits; there were more flower or seed resources and higher numbers of bees or birds on AES areas managed by trained farmers relative to untrained farmers (summarized in Dicks et al. [2013*a*]). It has been repeatedly demonstrated that farmer field schools, common in low and middle income countries, enhance uptake of beneficial integrated pest management practices, although the schools do not seem to spread practices through the farming community beyond the attendees (Waddington et al. 2014). Results-oriented AES is another approach with potential to generate long-term positive behavioral change by providing incentive for farmers to improve their skills (Burton & Schwarz 2013).

## Learning from the European experience

Almost everywhere in the world except Europe, Australia, and New Zealand, cultivated farmland is still expanding and natural habitats continue to be lost. Even if further conversion to farmland can be stopped, there is strong evidence that the agricultural matrix between areas of natural habitat is used by many wild species and holds important resources for some (Attwood et al. 2009; Mendenhall et al. 2014). In this context, policies such as AES that encourage farming practices less harmful to wildlife could become a standard part of conservation policy more widely in the coming decades.

Conservation programs that provide incentives directly to farmers to protect and manage land for biodiversity are not unique to Europe. Other parts of the world with intensive agriculture have comparable schemes, such as the Conservation Reserve Program, the Environmental Quality Incentives Program, and the Wetlands Reserve Program in the United States (Lambert et al. 2007) and the Landcare and Conservation Reserve Program in Australia (Hajkowicz 2009). The Australian program differs from European AES in that it aims to restore natural habitat (grasslands, shrublands, forests) on farmland rather than maintain the farmland itself. Compared with the amount of research in Europe, there is little information on the effectiveness of the Australian and U.S. schemes (but see, e.g., Riffell et al. [2008] and Attwood et al. [2009]). So what has been learned in Europe that could be applied in the rest of the world?

Research over the last 20 years shows that European AES have been generally beneficial for farmland biodiversity, leading in the majority of cases to a moderate increase in numbers of species present. There are suggestions that they have slowed the loss of farmland biodiversity in some countries (Carvalheiro et al. 2013).

Europeans have learned that the structure of the surrounding landscape and the degree of ecological contrast between land under schemes and the immediate surroundings are important moderators of this effectiveness. This understanding creates an opportunity to target AES toward areas where they are most likely to be effective, in intensively farmed landscapes of intermediate complexity, where they generate high ecological contrast by providing resources that are limited in the surroundings or potentially by buffering protected areas (although this is untested).

Europeans have also learned that AES are an expensive way to do conservation. As a policy tool, they are complex. It is not easy to improve their effectiveness in response to new research because they have to be easy to implement, feasible on a large scale, and palatable to farmers. As a result, it could be argued that AES should only be employed in parts of the world, such as Europe, where a high proportion of the unique or declining biodiversity depends directly on farmland or farming activities.

## Supporting Information

Characteristics of AES in Europe (Appendix S1); a summary of reviews of effectiveness of European AES (Appendix S2); a summary information for each observation included in the meta-analyses (Appendix S3); the funnel plot, regression test results, and fail-safe number (Appendix S4); a summary table of meta-analysis results (Appendix S5); country codes (Appendix S6); and further discussion of cost-effectiveness of AES (Appendix S7) are available online. The authors are solely responsible for the content and functionality of these materials. Queries (other than absence of the material) should be directed to the corresponding author.
