# Supplementary material for: The role of agri-environment schemes in conservation and environmental management
Source: Conserv Biol. 2015 May 21;29(4):1006–16. doi: 10.1111/cobi.12536 (PMC4529739; doi:10.1111/cobi.12536)
Supplement: Supplementary file 1 — Characteristics of AES in Europe (Appendix S1); a summary of reviews of effectiveness of European AES (Appendix S2); a summary information for each observation included in the meta-analyses (Appendix S3); the funnel plot, regression test results, and fail-safe number (Appendix S4); a summary table of meta-analysis results (Appendix S5); country codes (Appendix S6); and further discussion of cost-effectiveness of AES (Appendix S7) are available online. The authors are solely responsible for the content and functionality of these materials. Queries (other than absence of the material) should be directed to the corresponding author. [file cobi0029-1006-sd1.pdf]

Supporting Information

Appendix S1. Characteristics of agri-environment programmes in European countries until the year 2013. UAA, Utilized Agricultural Area; AEP, agri-environment programme; AES, agri-environment scheme. Many countries have described some of their schemes as ‘horizontal’, which refer to broad and shallow, or lower tier schemes. UAA (2007) and area with AES (2012) data were derived from EU (2014). UAA (2012) data for Croatia, Norway and Switzerland were derived from FAOSTAT (URL: http://faostat3.fao.org). Further information on agri-environment programmes of EU countries for 2007-2013 can be found in the Rural Development Programmes of each member states at the EU website of European Network for Rural Development (URL: http://enrd.ec.europa.eu/enrd-static/policy-in-action/rural-development-policy-overview/national-and-regional-programmes/en/national-and-regional-programmes_en.html).

**Austria**. (UAA: 3 189 110 ha; area with AES: 2 181 453 ha; AEP since 1995, previous programme outside the EU-context since 1972). The Austrian AES ÖPUL is a horizontal program that aims at a full coverage of the Austrian agriculture. Its focus is on the conservation of water, soil, climate, biodiversity and cultural landscapes. ÖPUL consists (in the version valid since 2007) in 29 measures, which are mostly offered throughout the entire country. In 2013, the scheme covered 91% of the Austrian UAA (except alpine pastures) and 109 000 agricultural businesses participated. *Highest uptake*: in 2013 (total uptake in Austria: 529 M€) environmental management (20.1%) and organic farming (18.5%). *Source*: Anonymous (2014a). *Information provider*: Stefan Schindler.
**Belgium**. (UAA: 1 374 430 ha; area with AES: 199 050 ha; AEP since 1994). Each of the three regions of Belgium has its own AEP. In the two regions with significant agricultural activities, among all schemes, some aim to preserve native breeds and elements of the ecological network and landscape (e.g. hedges, ponds, natural grasslands). Others aim to reduce fertilizers and pesticides inputs by limiting the quantities used in cereal crops or by keeping a low stocking rate, or to limit their leaching by installing a winter ground cover. A third main aim is to host natural flora and fauna on a portion of cultivated fields (e.g. flower strips, protection of river banks, beetle banks). *Source*: Anonymous (2005). *Information provider*: Pierre Rasmont and Sarah Vray.
**Bulgaria**. (UAA: 3 050 740 ha; area with AES: 388 888 ha; AEP since 2007). The main aims of the AEP, which can be applied across Bulgaria, are: maintenance of high nature value (HNV) arable land, organic farming, landscape characteristics, traditional farming and protection of soil and waters. The schemes include maintenance of HNV arable habitats for birds, with several zonal schemes for globally threatened bird species. *Highest uptake*: Two thirds of the AES budget went towards “protection of soil and waters” mainly in 2013. *Source*: Anonymous (2013a). *Information provider*: Edita Difova.

**Croatia**. (UAA: 1 327 730 ha; AEP since 2013). Croatia joined the EU in 2013. However, there was a comprehensive pre-accession Rural Development Programme that Croatia implemented until the end of the programming period 2007–2013. The pilot agri-environment programme were designed to address two major problems: 1.) Decline of landscape, habitats and species diversity due to loss of agricultural land, notably grassland; 2.) Environmental degradation caused by inappropriate agriculture practices including high consumption of fertilisers and pesticides, notably on arable and permanent crops. It included three site specific measures: preventing further natural succession on species-rich grasslands (Velebit Nature Park); restoring and maintaining wetland grassland (Lonjsko Polje Nature Park) and an arable farming pilot measure (Zagrebačka County). *Source*: IPARD (2013).
**Cyprus**. (UAA: 146 000 ha; area with AES: 24 028 ha; AEP since 2004). The Agro-environmental Commitments consists of eight sub-measures: 1.) Reduction of chemical weeding in vineyards; 2.) Reduction of pesticides and fertilisers in potatoes; 3.) Reduction of pesticides and chemical weeding in citrus fruits; 4.) Increasing soil fertility and quality as well as reduction of the use of pesticides and fertilisers in arable crops; 5.) Preservation of traditional vineyard varieties and endangered species; 6.) Preservation of landscape with traditional trees and bushes, such as almond trees, carob trees, hazelnut trees and Rosa damaskina; 7.) Encouraging organic production both in animal and plant sector; 8.) Preservation of habitats necessary for the reproduction of wild fauna, provision of the necessary biomass for wild birds and mammals feeding. *Source*: RDP Cyprus (2013).
**Czech Republic**. (UAA: 3 518 070 ha; area with AES: 1 069 741 ha; AEP since 2004). AES in the Czech Republic has 3 sub-schemes, which are divided into–1. Environmentally friendly farming methods (organic farming, integrated farming), 2. Grassland maintenance (with special titles targeted at specific priority grassland habitats), 3. Landscape Care (conversion of arable to grassland, cover crops, wildlife strips). The basic condition for receiving payments under the AES is closing five-year commitment. *Highest uptake*: Organic farming ca. 25 % AES budget for 2007–2013; Pastures ca. 20 % AES budget for years 2007–2013. *Source*: Černá et al. (2007). *Information provider*: Jarmila Kostiuková and Jana Dandová.
**Denmark**. (UAA: 2 662 590 ha; area with AES: 160 817 ha; AEP since 1992, previous schemes under regulation 797/85 since 1990). Danish agri-environmental schemes have following main targets: First, to avoid eutrophication of water bodies. These schemes are both horizontal (reduction of fertilizer and pesticides, conversion to organic farming) and geographically specific (mandatory buffer zones are compensated, and establishment and up-keeping wetlands are subsidised). You may also see subsidies to energy crops partly in this category, as they are perennial. Second, to support biodiversity. These schemes both horizontal (subsidy for grazing and mowing of various types of grassland and nature areas) or targeted at Natura 2000 areas, such as subsidies for clearing of areas for grazing, or establishment and up-keeping of natural hydrology. *Highest uptake*: Various forms of grassland schemes (grazing, mowing, extensive use), which compose 92 % of the non-organic schemes. *Source*: Anonymous (2014b). *Information provider*: Pia Frederiksen and Gregor Levin.

**Estonia**. (UAA: 906 830 ha; area with AES: 600 041 ha; AEP since 2004). Estonian AEP consists of five sub-measures which can be applied across Estonia. The objectives of the AEP are to: promote the implementation and continuous use of environmentally friendly management methods in agriculture; preserve and increase biological and landscape diversity; help farmers act in an environmentally favourable way whilst maintaining an adequate income; increase environmental awareness. Three of the sub-measures are horizontal schemes: organic farming, environmentally friendly management (basic and additional scheme) and maintenance of semi-natural habitats. In addition, there are schemes to support growing one local plant variety and keeping animals of four local endangered breeds. *Highest uptake*: Environmental Friendly Management has the *Highest uptake* – 57% from AEP budget in 2012 (about 77% from AEP farmland area in 2012). *Source*: Anonymous (2008). *Information provider*: Riho Marja.
**Finland**. (UAA: 2 292 290 ha; area with AES: 2 181 247 ha; AEP since 1995). The Finnish AEP comprises two tiers, basic and additional, and a special package. The lower tier is a prerequisite to all participants in the AEP, includes basic conditions for environmentally friendly production (e.g., soil nutrient analysis, buffer strips; in 2009, environmental fallow was added). On top of the basic, participants must choose at least one or two (depending on the region) measures from the additional package (e.g. more stringent fertilization limits, winter cover options). The special package includes among others organic production, management of semi-natural grasslands, traditional breeds and varieties. *Highest uptake*: 90 % of the farmers (92 % of the UAA) had AEP contracts (basic level) in 2010. *Source*: Anonymous (2013b). *Information provider*: Irina Herzon.
**France**. (UAA: 27 476 930 ha; area with AES: 6 000 000 ha; AEP since 1992, previous schemes under regulation 797/85 since 1989). The French AEP includes national, regional and more locally focused measures. Within the period 2007-2013, the schemes aimed to preserve biodiversity and water resource quality. The schemes were defined at national or regional scales (horizontal schemes; e.g. organic management, maintenance of extensively managed grasslands, mixed-farming systems, crop rotations), and can be adapted locally (zonal schemes; e.g. grassland managed for bird nesting protection, mountainous grassland maintenance through pastoralism). *Source*: Anonymous (2012a). *Information provider*: Aliette Baillod.
**Germany**. (UAA: 16 931 900 ha; area with AES: 5 039 302 ha; AEP since 1992, previous schemes under regulation 797/85 since 1985). Each of the 16 federal states of Germany has its own AEP resulting in a variety of different measures. German AES can be divided in two main types. First, schemes aimed at making agricultural production more environmentally friendly (horizontal schemes; e.g. organic management, grassland extensification, flower strips) and second, schemes aimed at preservation of specific biotopes or species (zonal schemes; e.g. management of calcareous grasslands, orchard meadows or bird resting areas). All federal states provide additional, but different AES without the co-funding of the EU. *Highest uptake*: Examples of uptake for two states: organic management in Lower Saxony and Bremen (32% of AEP budget on 19% of AEP area) and organic management in Bavaria (23% of AEP budget). *Source*: Thomas et al. (2009). *Information provider*: Péter Batáry, Sebastian Klimek and Christian Wagner.

**Greece**. (UAA: 4 076 230 ha; area with AES: 500 000 ha; AEP since 1995, previous schemes under regulation 797/85 since 1986). Greece applied four schemes for the whole country (organic farming and organic animal husbandry, conservation of indigenous animal breeds and conservation of plants), five schemes for Natura 2000 wetland sites, two for landscapes, two for landscape features, and two for intensive practices (set aside and fertilization reduction). *Highest uptake*: The most popular measure is the organic scheme (36% of AEP budget). *Source*: Anonymous (2014c). *Information provider*: Theodora Petanidou.
**Hungary**. (UAA: 4 228 580 ha; area with AES: 1 153 910 ha; AEP since 2004). Hungarian AES can be divided in two main types. First, there are schemes aimed at making agricultural production more environmentally friendly (horizontal schemes): Wetland scheme, Grassland scheme, Organic production scheme, Integrated production scheme, Agri-environmental basic scheme. Second, there are zonal (regional) schemes for areas with low production potential but significant natural value. Scheme measures vary between areas and include conversion of arable land to grassland, use of extensive farming methods, maintenance of endangered breeds, habitat restoration and development, landscape reconstruction measures and provision of favourable condition for important bird species (e.g. great bustard and red-footed falcon). *Highest uptake*: In 2012 the integrated production scheme had the highest uptake in terms of area (52% AEP area). *Source*: Anonymous (2009), NHRDP (2011). *Information provider*: Péter Batáry, Anikó Kovács-Hostyánszki.
**Ireland**. (UAA: 4 139 240 ha; area with AES: 2 526 950 ha; AEP since 1994). The Irish Agri-Environment Option Scheme contains three objectives; one is contribution to halting biodiversity decline. There are actions at three levels: Primary Environmental Actions (Species rich grassland, Traditional hay meadows, Establishment & Maintenance of Habitats, Wild Bird Cover); Complementary Actions (e.g. Riparian Margins, Traditional Orchards, Coppicing hedgerows); Additional Actions (e.g. Planting of new hedgerows, Arable Margins, Minimum Tillage). *Source*: Anonymous (2010a). *Information provider*: John A. Finn.
**Italy**. (UAA: 12 744 200 ha; area with AES: 2 356 962 ha; AEP since 1994/5). Each of the 20 Italian regions has its own rural development plans resulting in a variety of different measures. The large majority of schemes are aimed at making agricultural production more sustainable (horizontal schemes such as organic management), while schemes aimed at preservation of specific biotopes or species are rarer (e.g. conservation of wetlands or dry grasslands). *Highest uptake*: Scheme uptake of the different regions does not present a geographical trend. The three regions with the highest participation to the schemes are Bolzano (41% of AEP budget), Basilicata (32% of AEP budget), and Sicily (31% of AEP budget). *Source*: Anonymous (2014d). *Information provider*: Lorenzo Marini.
**Latvia**. (UAA: 1 773 840 ha; area with AES: 235 050 ha; AEP since 2004). There were four AES sub-measures available in Latvia. One scheme ("Maintenance of Biological Diversity in Grasslands") was zonal and aimed at preventing further loss and degradation of semi-natural grasslands and was the only truly biodiversity oriented AES in the country. The rest of the schemes ("Development of Biological Farming", "Introducing and Promoting Integrated Horticulture" and "Stubble Field in Winter Period" were horizontal and aimed at promoting certain environment friendly farming practices, including reduction of use of agrochemicals and reduction of nutrient leakage. *Highest uptake*: Development of Biological Farming (74% of AES budget). *Source*: Anonymous (2013c). *Information provider*: Ainars Aunins.

**Lithuania**. (UAA: 2 648 950 ha; area with AES: 251 837 ha; AEP since 2004). The Lithuanian AEP comprises two major groups of schemes. The first is for environmental friendly/sustainable and extensive agricultural production (e.g. organic and sustainable management, expansion of grasslands, increasing crop diversification). The second has a more explicit conservation focus in agricultural areas (e.g. protection of water, soil, biodiversity and landscape, Natura 2000 habitat, protective zones close water bodies, wetlands and melioration programmes, afforestation). *Source*: Anonymous (2013d). *Information provider*: Ligita Baležentienė.
**Luxembourg**. (UAA: 130 880 ha; area with AES: 118 335 ha; AEP since 1996). Luxembourg has several types of agri-environmental measures. Payments are for measures such as 1.) promotion of organic agriculture; 2.) management of agricultural landscape (e.g. maintenance of permanent grassland, adequate arable land fertilization); 3.) environmental friendly practices (e.g. delayed grass mowing, diverse crop rotation) and others (e.g. set-aside land, maintenance of traditional orchards). *Highest uptake*: management of agricultural landscapes (about 80% of all AES budget). *Source*: Anonymous (2007a).
**Malta**. (UAA: 10 330 ha; area with AES: 2 042 ha; AEP since 2004). The AES contain the following sub-measures: 1.) use of the environmentally friendly plant protection methods in vineyards; 2.) traditional crop rotation including the cultivation of sulla (Hedysarum coronarium); 3.) low input farming; 4.) suppress the use of herbicides in vineyards and fruit orchards; 5.) establishment and maintenance of conservation buffer strips; 6.) conservation of rural structures providing a natural habitat for fauna and flora; 7.) providing a healthy forage area for bees; 8.) organic farming. *Source*: RDP Malta (2013).
**Netherlands**. (UAA: 1 914 330 ha; area with AES: 228 303 ha; AEP since 1992, previous schemes partly under regulation 797/85 and partly outside the EU-context since 1981). The Dutch AEP has a variety of schemes targeting meadow birds, farmland passerines, European Hamster *Cricetus cricetus*, wintering geese, grassland flora and arable flora. Most schemes aim to promote the targeted species groups by prescribing measures that extensify farming activities. Examples include delaying first seasonal activities for meadow birds, reducing or prohibiting agro-chemical use for flora, planting wild bird seed mixtures for wintering farmland passerines or providing early and late season cover for European Hamster. *Highest uptake*: With about 60% of the total area covered by agri-environment schemes, meadow bird schemes are most popular in terms of uptake. *Source*: Anonymous (2010b). *Information provider*: David Kleijn.
**Norway**. (UAA: 991 700 ha; area with AES: 990 200 ha; AEP since 1990). There are AEP-schemes on national, regional and community level. The schemes aim to reduce the effect of agricultural practice on the environment, to preserve specific landscapes, biotopes, agriculural practices, grazing, organic farming, and to reduce pollution etc. The biggest scheme, the acreage- and cultural landscape scheme, takes up around 80 percent of the total AEP-budget. On a regional level there are two schemes directed at preserving specific environmental and cultural landscape qualities, to reduce water-pollution, contribute to biological diversity and public access to areas of recreational value. On a community level, there is a scheme directed towards preserving specific nature and cultural heritage elements and reduction of pollution from agriculture. *Highest uptake*: 98 percent of all farmers (2013) were obliged to take up the acreage- and cultural landscape scheme. The percentage was lower for other schemes. *Source*: Anonymous (2012b). *Information provider*: Oddmund Hjukse and Agnar Hegrenes.

**Poland**. (UAA: 15 477 190 ha; area with AES: 2 048 430 ha; AEP since 2004). Agri-environment schemes in 2007-2013 included nine projects (packets) divided into 49 variants: Sustainable Agriculture, Organic farming, Extensive permanent grassland, Protection of endangered bird species and habitats outside Natura 2000 sites, Protection of endangered bird species and habitats in Natura 2000 areas, Preservation of endangered plant genetic resources in agriculture, Preservation of endangered animal genetic resources in agriculture, Protection of soil and water, Buffer zones. *Source*: Brodzińska (2009). *Information provider*: Piotr Tryjanowski.
**Portugal**. (UAA: 3 472 940 ha; area with AES: 954 134 ha; AEP since 1994). The Portuguese Rural Development Plan includes two main AES. One scheme - Enhancement of production methods - is applied horizontally and aimed at promoting the sustainable development of rural areas, it supports: (i) organic farming and integrated production, (ii) conservation of traditional livestock breeds, (iii) conservation and improvement of genetic resources, including local varieties of plants and animal breeds, and (iv) soil conservation, in particular through use of direct seeding. The other scheme - Integrated Territorial Interventions - is zonal and addresses the conservation of biodiversity and cultural landscapes in Natura 2000 areas and in the Douro Wine region. *Highest uptake*: Organic farming and integrated production was implemented on 333 059 ha. 51% of these farms were in the North region and 61% of the area was located in Alentejo. *Source*: MAMAOT (2012). *Information provider*: Vânia Proença.
**Romania**. (UAA: 13 753 050 ha; area with AES: 1 840 559 ha; AEP since 2007). There is one AEP for the whole country, but different measures are spatially restricted. The largest measure in terms of budget and extent is focussed on High Nature Value (HNV) grasslands and arable land: in 2007-2013 the eligible area was 2.4 million ha (18 % UAA), this is likely to increase from 2014 onwards. There is also an "add-on" package for the use of traditional cultivation methods (e.g. hand scything, horse ploughing), which are still relatively widespread in the country. Further measures focus on the habitats of species of conservation concern (e.g. *Crex crex*, *Maculinea spp*.) and green cover crops. *Highest uptake*: HNV package with 1.11 million ha within the measure in 2012. *Source*: MARD (2014). *Information provider*: Laura M. E. Sutcliffe.
**Slovakia**. (UAA: 1 936 620 ha; area with AES: 357 175 ha; AEP since 2004). The Rural Development Programme (RDP) 2007-2013 includes 10 agri-environmental measures: Basic scheme; Erosion prevention on arable land; Erosion prevention in vineyards; Erosion prevention in orchards; Arable land grassing; Integrated production; Ecological agriculture; Protection of biotopes of semi-natural and natural grasslands; Protection of biotopes of selected birds species; Breeding and maintenance of threatened animal species. *Highest uptake*: Besides the basic scheme (300 000 ha), the largest area was supposed for Protection of biotopes of selected birds species (261 000 ha), Organic farming (150 000 ha), Erosion prevention (100 000 ha) and Protection of biotopes of semi-natural and natural grasslands (96 000 ha). *Source*: Baránková et al. (2010). *Information provider*: Lubos Halada.

**Slovenia**. (UAA: 488 770 ha; area with AES: 217 749 ha; AEP since 2004). The measures are horizontal and intended for all farmers in Slovenia. The measure of compensatory allowances may be applied only for areas designated under this programme as Less Favoured Areas. Certain spatial restrictions apply also for some specific agri-environmental sub-measures, which are protection regimes or management requirements for the preservation of individual habitat types. In the period 2007-2013 there were 24 measures divided into three groups according to the objectives to be achieved by individual measures: Reducing the negative impacts of agriculture on the environment, Conservation of natural resources, biodiversity, soil fertility and traditional cultural landscape, Conservation of Protected Areas. *Highest uptake*: *Highest uptake* is in the most agricultural areas, of the north east. However, programs dedicated to conservation on grassland are much more present in Western part of Slovenia. *Source*: Anonymous (2007b). *Information provider*: Mitja Kaligarič and Jure Čuš.
**Spain**. (UAA: 24 892 520 ha; area with AES: 5 091 250 ha; AEP since 1993). In Spain, AEPs are implemented by the 17 individual regional governments and the National Rural Network by the Spanish government. Horizontal schemes and specific measures, responding to different regional situations are included in each AEP. The development of the AEPs is coordinated by the Spanish government to ensure the consistency of the Spanish strategy for rural development throughout the territory. *Highest uptake*: Extremadura is the region of Spain that has more surface with agri-environment payments (9% of the UAA, which represents 31% of the total surface with AEP). *Source*: Anonymous (2012c). *Information provider*: F. Xavier Sans.
**Sweden**. (UAA: 3 118 000 ha; area with AES: 1 907 589 ha; AEP since 1995, previous schemes outside the EU-context since 1986). The Swedish RDP 2007-2013 consists of four ‘axes’, of which one focuses on ‘enhancing environment and landscape’ (axis 2). The main objectives of this axis are to conserve biodiversity, to maintain naturally and culturally valuable and varied landscapes, and to minimize pesticide use and nutrient leaking. Most AEP’s in axis 2 are zonal or combine zonal and horizontal schemes. Similarly to the previous period, AEP’s aiming at maintaining open landscapes and conservation of semi-natural grasslands and cultural elements are most popular. *Highest uptake*: Perennial ley farming (44% of AEP area), organic farming (21%) and maintenance of semi-natural grasslands (20%). *Source*: SJV (2014). *Information provider*: Juliana Dänhardt.
**Switzerland**. (UAA: 1 528 700 ha; area with AES: 129 889 ha; AEP since 1993). Farmers need to farm at least 7% of their land according to the guidelines for ecological focus areas (EFA) in order to qualify for subsidies (this is a ‘cross-compliance’ mechanism, set at 3.5% for horticultural farms). They can choose from a suite of 16 different EFA types. For eight EFA types, criteria for ecological quality have been defined (based on indicator plants and structural diversity). If quality criteria are met, the farmer is entitled to bonus payments (result oriented scheme). In addition, farmers as a group can propose a project in which they formulate measures to increase the share, quality and connectivity of EFA in order to promote selected target species. Participation in such a project is remunerated by bonus payments. *Highest uptake*: The share of EFA tends to be higher in mountain areas: 28% of UAA on average in the highest mountain farming region. *Source*: Anonymous (2014d). *Information provider*: Felix Herzog.

United Kingdom. (UAA: 16 130 490 ha; area with AES: 5 312 613 ha; AEP since 1992, previous schemes under regulation 797/85 since 1987). England and Wales both had two-tier schemes. Entry Level Stewardship (ELS, in England, now ended) or Glastir Entry (Wales) were flexible and untargeted, allowing farmers to select from a wide variety of management actions to meet a threshold score. Agreements were for five years. Higher Level Stewardship (HLS) or Glastir Advanced agreements were carefully targeted for specific biodiversity objectives, implemented with advice from experts on priority sites selected at regional level. HLS agreements lasted 10 years. Scotland had a single tier, Rural Stewardship Scheme, in which farmers could select from a range of objective-driven management options, based on a whole farm environmental audit. Agreements lasted at least five years (now ended). Northern Ireland has a single tier, whole-farm Countryside Management Scheme, open to all farmers. *Highest uptake*: England had 68% of farmland under Entry Level Stewardship in 2012. 16% of English farmland was under Higher Level Stewardship in 2012, but most was also in the Entry Level scheme. *Source*: Defra (2013). *Information provider*: Lynn V. Dicks.

Literature Cited

Anonymous. 2005. Agriculture and Rural Development thematic site on EUROPA, 2005. Evaluation des mesures agro-environnementales AGRI/G4/2004, Annexe 8: Etude nationale Belgique. Oréade-Brèche Environnement & Développement.

Anonymous 2007a. Programme de développement rural du G.-D. de Luxembourg (2007-2013). Pour une campagne vivante. Le Gouvernement du Grand Duché de Luxembourg Ministère de l'Agriculture, de la Viticulture et du Développement rural. Version approuvée le 19 octobre 2007 par la Commission Européenne (CCI 2007 LU 06 RPO 001).

Anonymous. 2007b. National Strategy Plan for Rural Development 2007- 2013. Ministry of Agriculture, Forestry and Food. Republic of Slovenia.

Anonymous. 2008. Estonian Rural Development Plan 2007-2013. Ministry of Agriculture, Estonia.

Anonymous. 2009. Hungarian Parliamentary Regulation on the National Agri-environment Program 2009-2014. 96/2009. (XII. 9.)

Anonymous 2010a. Terms and Conditions of the agri-environment options scheme (AEOS) and the NATURA 2000 scheme. URL: http://www.agriculture.gov.ie/media/migration/farmingschemesandpayments/ruralenvironmentprotectionschemereps/ruralenvironmentprotectionschemereps/aeosschemeforms/AEOSNaturaTandC300913.pdf

Anonymous 2010b. Index Natuur en Landschap Onderdeel Agrarische beheertypen. URL: http://www.portaalnatuurenlandschap.nl/assets/Index-Agrarisch-2011.pdf

Anonymous. 2012a. French Agricultural Ministry, 2012. Les mesures agroenvironnementales. Wagram Editions.

Anonymous. 2012b. Nasjonal miljøplan. Statens landbruksforvaltning. Rapport nr. 23/2012. (National environmental programme 2012. Norwegian Agricultural Authority).

Anonymous. 2012c. Marco nacional de Desarrollo Rural 2007-2013. Ministerio de Agricutlura, Alimentación y medi Ambiente. Madrid. URL: http://www.magrama.gob.es/es/desarrollo-rural/temas/programas-ue/Marco_Nacional_mayo2012_tcm7-9907.pdf

Anonymous. 2013a. Bulgarian Rural Development Plan 2007-2013. MAF, Bulgaria.

Anonymous. 2013b. The Rural Development Programme for Mainland Finland 2007-2013. Finland.

Anonymous. 2013c. Rural Development Programme of Latvia 2007 – 2013. 12th edition. Latvian Ministry of Agriculture, Riga, 464 pp.

Anonymous 2013d. The progress of the implementation of the Lithuanian Rural Development Programme 2007–2013 in the year 2012. Republic of Lithuania, Agricultural Ministry, Vilnius, 2013/197.

Anonymous. 2014a. Bundesministerium für Land- und Forstwirtschaft, Umwelt und Wasserwirtschaft. Grüner Bericht 2014. Bericht über die Situation der österreichischen land- und Forstwirtschaft. 55. Auflage, Wien. URL: http://www.gruenerbericht.at/cm3/download/finish/82-gruener-bericht-oesterreich/1392-gruener-bericht-2014/0.html

Anonymous. 2014b. Vejledning om tilsagn til 5-årige miljø- og økologiordninger og miljøvenlige jordbrugsforanstaltninger. Ministeriet for Fødevarer, Landbrug og Fiskeri. København.

Anonymous. 2014c. Strategic Targets for Agricultural Development And Restructuring of the Countryside. URL: http://www.minagric.gr/index.php/el/

Anonymous. 2014d. Rete Natura Rurale. Ricognizione Programmi di sviluppo rurale EUROPA Misura 214 “Pagamenti Agroambientali”. Ministero delle Politiche Agricole Alimentari e Forestali.

Anonymous. 2014d. Swiss Confederation. Bundesamt für Landwirtschaft BLW. Ökologie. URL: http://www.blw.admin.ch/dokumentation/01535/01544/index.html?lang=de

Baránková, Z., L. Halada, Z. Izakovičová, and B. Šatalová. 2010. Influence of indicators for policy development in Agrienvironmental programmes in the Slovak Republic. Institute of Landscape Ecology SAS, Bratislava.

Brodzińska K. 2009: Kierunki i perspektywy rozwoju programu rolnośrodowiskowego w Polsce po 2013 roku. Woda-Środowisko-Obszary Wiejskie, Instytut Melioracji i Użytków Zielonych w Falentach. t. 9, z. 3(27), s. 5-18.

Černá, M., B. Fišer, E. Potočiarová, and A. Vejvodová. 2007. Agroenvironmentální opatření České republiky 2007–2013 [Agri-environmental schemes in the Czech Republic 2007–2013]. MoE CR, NCA CR, MoA CR, Prague, Czech Republic.

Defra 2013. Environmental Stewardship Handbooks, edition 4. URL: https://www.gov.uk/environmental-stewardship

EU. 2014. Rural developement in figures. Financial and physical indicators. URL: http://enrd.ec.europa.eu/enrd-static/policy-in-action/rural-development-policy-in-figures/rdp-monitoring-indicator-tables/financial-and-physical-indicators/en/financial-and-physical-indicators_en.html

IPARD 2013. IPARD Programme 2007-2013. Agriculture and Rural Development Plan. Ministry of Agriculture. Zagreb. URL: http://www.mps.hr/ipard/UserDocsImages/dokumenti/IPARD/IPARD%202013/IPARD%20VI%202013%20EN%20FINAL.pdf

MAMAOT (2012) Programa de Desenvolvimento Rural: Continente 2007-2013. Revisão 09 – Janeiro 2012. MAMAOT, Lisboa. URL: http://www.proder.pt/conteudo.aspx?menuid=366

MARD. 2014. Raport anual de progrese privind implementarea programului naţional de dezvoltare rurală în România în anul 2013 [Annual progress report for the implementation of the national rural development program in Romania in 2013]. Ministry of Agriculture and Rural Development, Bucharest.

NHRDP 2011. New Hungary Rural Development Programme. Budapest. URL: http://enrd.ec.europa.eu/enrd-static/fms/pdf/BA7A2748-FBA5-23D9-8FC1-A61716C5AD57.pdf

RDP Cyprus 2013. Description of rural development programme 2007-2013. Cyprus. URL: http://www.moa.gov.cy/moa/da/da.nsf/A9AC8C327C7C318CC22579AD0028B11B/$file/CyprusRDPSummary2007_2013.pdf

RDP Malta 2013. Rural Development Programme for Malta 2007-2013. Ministry for Sustainable Development, the Environment and Climate Change. Malta. URL: https://secure2.gov.mt/MRRA-MA/file.aspx?f=379

SJV. 2014. Årsrapport landsbygdsprogrammet 2013, SJV Rapport 2014:17.

Thomas, F., K. Denzel, E. Hartmann, L. Rainer, and K. Schmoock. 2009. Kurzfassungen der Agrarumwelt- und Naturschutzprogramme. Bundesamt für Naturschutz, Bonn, Germany.

Appendix S2. Summary of reviews of effectiveness of European agri-environment schemes.

Years	*Topic*	*Conclusions*	*Reference*
1983-2000	Overview of state of AES in 26 European countries	AES varied markedly between countries. Highest uptake of AES in extensive agricultural areas. Research studies only in 6 countries with a dominance of UK and NL. Majority of studies was inadequate to assess reliably the effectiveness of the schemes. Nevertheless more than the half of examined species (groups) demonstrated increases in species richness or abundance compared with controls.	Kleijn and Sutherland (2003)
1986-2002*	Meta-analysis on the effects of organic management on biodiversity	Organic farming had on average 30% higher species richness and 50% more organisms than conventional farming systems (ca. three-quarters of all data from Europe), but results were highly variable between studies and organism groups. They proposed that the effects of organic farming are larger in intensively managed landscapes than in small-scale diverse landscapes with many non-crop biotopes.	Bengtsson et al. (2005)
1994-2008*	Meta-analysis on landscape moderation effect on effectiveness of agri-environment management (AEM)	AEM significantly increased species richness and abundance of plants and animals (ca. 80% of all data from Europe). In croplands, species richness but not abundance was significantly enhanced in simple but not in complex landscapes. In grasslands, AEM effectively enhanced species richness and abundance regardless of landscape context. They concluded that AEM should be adapted to landscape structure.	Batáry et al. (2011)
1994-2011*	Meta-analysis on the landscape moderation effect on AES effectiveness in case of pollinators (complementing Batáry et al. 2011)	They found that the ecological contrast in floral resources created by schemes drives the response of pollinators to AES (only European studies). This response is moderated by landscape context and farmland type, with more positive responses in croplands (vs. grasslands) located in simple (vs. cleared or complex) landscapes.	Scheper et al. (2013)
1986-2011*	Meta-analysis on benefits of organic farming to biodiversity (also updating Bengtsson et al. 2005)	Organic farming increased species richness by about 30% (ca. 84% of all data from Europe). This result was robust over the last 30 years of published studies and shows no sign of diminishing. Organic farming had a greater effect on biodiversity as the percentage of the landscape consisting of arable fields increased, i.e. in more intensively farmed regions.	Tuck et al. (2014)

*: based on years of publication of primary papers.

Literature Cited

Batáry, P., A. Báldi, D. Kleijn, and T. Tscharntke. 2011. Landscape-moderated biodiversity effects of agri-environmental management – a meta-analysis. Proceedings of the Royal Society B-Biological Sciences **278**:1894–1902.

Bengtsson, J., J. Ahnström, and C. Weibull. 2005. The effects of organic agriculture on biodiversity and abundance: a meta-analysis. Journal of Applied Ecology **42**:261–269.

Kleijn, D., and W. J. Sutherland. 2003. How effective are European agri-environment schemes in conserving and promoting biodiversity? Journal of Applied Ecology **40**:947–969.

Scheper, J., A. Holzschuh, M. Kuussaari, S. G. Potts, M. Rundlöf, H. G. Smith, and D. Kleijn. 2013. Environmental factors driving the effectiveness of European agri-environmental measures in mitigating pollinator loss – a meta-analysis. Ecology Letters 16:912–920.

Tuck, S. L., C. Winqvist, F. Mota, J. Ahnström, L. A. Turnbull, and J. Bengtsson. 2014. Land-use intensity and the effects of organic farming on biodiversity: a hierarchical meta-analysis. Journal of Applied Ecology **51**:746–755.

Appendix S3. Summary information for each observation included in the meta-analyses.
We split the data of a large EU project, called EASY, according to the study regions per country (observations from this project are marked by star). Source shows from which meta-analysis the data is coming (1: Batáry et al. 2011; 2: Scheper et al. 2013; 3: Tuck et al. 2014). Study year: for analysing the budget period, we considered always the last year of the studies. AES: I = in production scheme, O = out of production. N: sample size of AES plus control. g: Hedges' g. np var g: non-parametric variance estimate of Hedges' g.

Publication	*Source*	*Study year*	*Country*	*Organism*	*Habitat*	*AES*	*N*	*g*	*np var g*
Aavik & Liira 2010	3	2008	Estonia	Plants	Cereal	I	42	1.037	0.111
Aavik & Liira 2010	3	2008	Estonia	Plants	Cereal	I	42	0.074	0.111
Albrecht et al. 2007*	2	2004	Switzerland	Butterfly	Grassland	I	26	0.517	0.154
Albrecht et al. 2007*	1	2004	Switzerland	Hoverfly	Grassland	I	26	1.158	0.154
Albrecht et al. 2007*	1	2004	Switzerland	Solitary bee	Grassland	I	26	0.974	0.154
Albrecht et al. 2007b*	2	2003	Switzerland	Bees	Grassland	I	26	0.456	0.154
Albrecht et al. 2010*	2	2004	Switzerland	Bees	Grassland	I	48	1.271	0.083
Alvarez et al. 2001	1	1997	United Kingdom	Collembola	Cropland	I	22	0.347	0.188
Aude et al. 2003	3	2001	Denmark	Plants	Unspec	I	26	1.938	0.154
Aviron et al. 2009	2	2004	Switzerland	Butterflies	Cropland	O	150	0.811	0.027
Aviron et al. 2009	2	2004	Switzerland	Butterflies	Grassland	I	531	0.149	0.008
Aviron et al. 2010	2	2004	Switzerland	Butterflies	Cropland	O	43	2.375	0.088
Batáry et al. 2010	1	2008	Germany	Bird	Grassland	I	20	0.365	0.200
Batáry et al. 2010	1	2008	Germany	Bird	Cropland	I	20	0.491	0.200
Batáry et al. 2012	1	2008	Germany	Carabid	Grassland	I	20	0.432	0.200
Batáry et al. 2012	1	2008	Germany	Carabid	Cropland	I	20	0.763	0.200
Batáry et al. 2012	1	2008	Germany	Grasshopper	Grassland	I	18	0.180	0.222
Batáry et al. 2012	1	2008	Germany	Plant	Cropland	I	20	2.090	0.200
Batáry et al. 2012	1	2008	Germany	Plant	Grassland	I	20	0.949	0.200
Batáry et al. 2012	1	2008	Germany	Spider	Cropland	I	20	2.018	0.200
Batáry et al. 2012	1	2008	Germany	Spider	Grassland	I	20	1.031	0.200
Brittain et al. 2010	2	2006	Italy	Butterflies	Cropland	I	30	0.340	0.333
Brittain et al. 2010	2	2006	Italy	Solitary bees	Cropland	I	30	-0.113	0.333
Bruggissere et al. 2010	3	2005	Switzerland	Arthropods	Orchard	I	25	-0.357	0.250
Bruggissere et al. 2010	3	2005	Switzerland	Arthropods	Orchard	I	25	-0.324	0.250
Bruggissere et al. 2010	3	2005	Switzerland	Arthropods	Orchard	I	25	0.152	0.250
BTO 1995	3	1994	United Kingdom	Arthropods	Unspec	I	15	-0.355	0.268
Caballero-Lopez et al. 2010	3	2004	Spain	Plants	Cereal	O	8	4.549	0.500
Carvell et al. 2007	2	2004	United Kingdom	Bumblebees	Cropland	O	12	0.845	0.333
Carvell et al. 2007	2	2004	United Kingdom	Bumblebees	Cropland	O	12	3.560	0.333
Christensen et al. 1996	3	1987	Denmark	Birds	Mixed	I	8	0.480	0.500
Clough et al. 2007a*	1	2003	Germany	Carabid	Cropland	I	12	-0.096	0.333
Clough et al. 2007a*	1	2003	Germany	Carabid	Cropland	I	12	0.887	0.333
Clough et al. 2007a*	1	2003	Germany	Carabid	Cropland	I	14	-0.089	0.286
Clough et al. 2007a*	1	2003	Germany	Spider	Cropland	I	12	0.000	0.333
Clough et al. 2007a*	1	2003	Germany	Spider	Cropland	I	12	-0.412	0.333
Clough et al. 2007a*	1	2003	Germany	Spider	Cropland	I	14	0.201	0.286
Clough et al. 2007b*	1	2003	Germany	Rove beetle	Cropland	I	12	0.313	0.333

Appendix S3. Continued.

Publication	*Source*	*Study year*	*Country*	*Organism*	*Habitat*	*AES*	*N*	*g*	*np var g*
Clough et al. 2007b*	1	2003	Germany	Rove beetle	Cropland	I	12	0.388	0.333
Clough et al. 2007b*	1	2003	Germany	Rove beetle	Cropland	I	14	0.170	0.286
Concepción et al. 2008*	1	2003	Spain	Bee	Cropland	I	6	0.163	0.667
Concepción et al. 2008*	1	2003	Spain	Bee	Cropland	I	14	0.373	0.286
Concepción et al. 2008*	1	2003	Spain	Bee	Cropland	I	14	-0.238	0.286
Danhardt et al. 2010	3	2005	Sweden	Birds	Mixed	I	12	0.986	0.333
Danhardt et al. 2010	3	2005	Sweden	Birds	Mixed	I	12	-0.191	0.333
de Snoo et al. 1998	2	1992	Netherlands	Butterflies	Cropland	O	40	1.318	0.238
Diekötter et al. 2010	3	2007	Germany	Arthropods	Cereal	I	6	1.019	0.667
Diekötter et al. 2010	3	2007	Germany	Arthropods	Cereal	I	6	-1.167	0.667
Diekötter et al. 2010	3	2007	Germany	Arthropods	Cereal	I	12	-0.092	0.333
Dietschi et al. 2007	1	2003	Switzerland	Plant	Grassland	I	31	1.940	0.130
Döring et al. 2003	3	1999	Germany	Arthropods	Cereal	I	20	1.297	0.220
Döring et al. 2003	3	1999	Germany	Arthropods	Cereal	I	20	1.938	0.220
Döring et al. 2003	3	1999	Germany	Arthropods	Cereal	I	14	1.450	0.292
Döring et al. 2003	3	1999	Germany	Arthropods	Cereal	I	14	1.951	0.292
Ekroos et al. 2008	1	2003	Finland	Bumblebee	Grassland	I	55	0.539	0.092
Ekroos et al. 2008	1	2003	Finland	Butterfly	Grassland	I	55	-0.040	0.092
Ekroos et al. 2010	3	1998	Finland	Butterfly	Grassland	I	26	0.668	0.163
Ekroos et al. 2010	3	1998	Finland	Butterfly	Grassland	I	22	-0.271	0.259
Feber et al. 1996	3	1991	United Kingdom	Butterfly	Grassland	O	8	1.610	0.250
Feber et al. 1996	3	1991	United Kingdom	Butterfly	Grassland	O	8	0.600	0.250
Ekroos et al. 2010	1	1998	Finland	Butterfly	Grassland	I	26	4.105	0.163
Ekroos et al. 2010	1	1998	Finland	Butterfly	Grassland	I	22	1.357	0.259
Feber et al. 1998	1	1995	United Kingdom	Spider	Cropland	I	18	1.118	0.222
Feber et al. 2007	3	1996	United Kingdom	Spider	Cropland	I	20	0.906	0.200
Feber et al. 2007	3	1996	United Kingdom	Spider	Cropland	I	20	1.264	0.200
Fischer et al. 2011a	3	2008	Germany	Birds	Cropland	I	29	0.300	0.138
Fischer et al. 2011a	3	2008	Germany	Birds	Cropland	I	31	0.311	0.129
Fischer et al. 2011a	3	2008	Germany	Birds	Cropland	I	30	0.578	0.208
Fischer et al. 2011a	3	2008	Germany	Birds	Cropland	I	30	0.553	0.208
Fischer et al. 2011b	3	2008	Germany	Small mammals	Cropland	I	22	-0.078	0.182
Flohre et al. 2011	3	2008	Germany	Earthworms	Cropland	I	24	-0.796	0.167
Flohre et al. 2011	3	2008	Germany	Plants	Cropland	I	24	6.417	0.167
Fuentes-Montemayor et al. 2011	2	2008	United Kingdom	Macromoths	Cropland	O	36	0.141	0.125
Fuentes-Montemayor et al. 2011	2	2008	United Kingdom	Macromoths	Cropland and grassland	O	36	0.000	0.154
Fuentes-Montemayor et al. 2011	2	2008	United Kingdom	Macromoths	Cropland and grassland	O	36	0.414	0.125
Gabriel et al. 2010	3	2008	United Kingdom	Birds	Cereal	I	16	-0.787	0.250
Gabriel et al. 2010	3	2008	United Kingdom	Birds	Cereal	I	16	-0.779	0.250
Gabriel et al. 2010	3	2008	United Kingdom	Plants	Cereal	I	16	1.604	0.250
Gabriel et al. 2010	3	2008	United Kingdom	Plants	Cereal	I	16	2.018	0.250
Gabriel et al. 2010	3	2008	United Kingdom	Plants	Cereal	I	16	0.334	0.250
Gabriel et al. 2010	3	2008	United Kingdom	Plants	Cereal	I	16	0.184	0.250
Gabriel et al. 2010	3	2008	United Kingdom	Plants	Grass	I	16	0.803	0.250
Gabriel et al. 2010	3	2008	United Kingdom	Plants	Grass	I	16	0.256	0.250
Gabriel et al. 2010	3	2008	United Kingdom	Plants	Grass	I	16	0.289	0.250
Gabriel et al. 2010	3	2008	United Kingdom	Plants	Grass	I	16	0.020	0.250
Gabriel et al. 2006*	1	2003	Germany	Plant	Cropland	I	12	2.692	0.333
Gabriel et al. 2006*	1	2003	Germany	Plant	Cropland	I	12	3.054	0.333

Appendix S3. Continued.

Publication	*Source*	*Study year*	*Country*	*Organism*	*Habitat*	*AES*	*N*	*g*	*np var g*
Gabriel et al. 2006*	1	2003	Germany	Plant	Cropland	I	14	2.611	0.286
Galvan et al. 2009	3	2005	Netherlands	Microbes	Veg	I	10	0.198	0.400
Galvan et al. 2009	3	2005	Netherlands	Microbes	Veg	I	10	0.111	0.400
Gathmann et al. 1994	2	1990	Germany	Solitary bees	Cropland	O	8	0.000	0.500
Gathmann et al. 1994	2	1990	Germany	Solitary bees	Cropland	O	8	0.000	0.500
Genghini et al. 2006	1	1998	Italy	Bird	Cropland	I	41	1.161	0.105
Granqvist 1999	3	1998	Sweden	Plants	Cereal	I	16	0.146	0.250
Granqvist 1999	3	1998	Sweden	Plants	Grass	I	16	-0.540	0.250
Haenke et al. 2009	2	2006	Germany	Hoverflies	Cropland	O	14	1.791	0.286
Haenke et al. 2009	2	2006	Germany	Hoverflies	Cropland	O	14	3.135	0.286
Hawes et al 2010	3	2007	United Kingdom	Plants	Mixed	I	40	1.789	0.119
Hawes et al 2010	3	2007	United Kingdom	Plants	Mixed	I	40	1.667	0.119
Hodgson et al. 2010	2	2008	United Kingdom	Butterflies	Cropland	I	16	0.051	0.250
Hodgson et al. 2010	2	2008	United Kingdom	Butterflies	Grassland	I	16	-0.094	0.250
Hodgson et al. 2010	2	2008	United Kingdom	Butterflies	Cropland	I	16	0.000	0.250
Hodgson et al. 2010	2	2008	United Kingdom	Butterflies	Grassland	I	16	0.408	0.268
Hokkanen & Holopainen 1986	3	1984	Germany	Arthropods	Veg	I	7	1.213	0.583
Holzschuh et al. 2007*	1	2003	Germany	Bee	Cropland	I	12	2.792	0.333
Holzschuh et al. 2007*	1	2003	Germany	Bee	Cropland	I	12	1.537	0.333
Holzschuh et al. 2007*	1	2003	Germany	Bee	Cropland	I	14	0.654	0.286
Holzschuh et al. 2010*	2	2004	Germany	Solitary bees	Cropland	I	46	0.354	0.087
Hutton & Giller 2003	1	2000	Ireland	Dung beetle	Grassland	I	8	2.281	0.500
Hyvönen et al. 2003	3	1999	Finland	Plants	Cereal	I	105	3.373	0.039
Irmler 2003	1	1995	Germany	Carabid	Cropland	I	32	0.438	0.237
Jonasson et al. 2011	3	2009	Sweden	Arthropods	Cereal	I	60	1.308	0.075
Jonasson et al. 2011	3	2009	Sweden	Plants	Cereal	I	60	1.855	0.075
José-María & Sans 2011	3	2008	Spain	Plants	Cereal	I	30	1.254	0.133
José-María & Sans 2011	3	2008	Spain	Plants	Cereal	I	30	2.512	0.133
Kleijn et al. 1999	2	1998	Netherlands	Bees	Grassland	I	14	-0.086	0.286
Kleijn et al. 1999	2	1998	Netherlands	Butterflies	Grassland	I	14	-0.269	0.286
Kleijn et al. 1999	2	1998	Netherlands	Hoverflies	Grassland	I	14	0.171	0.286
Kleijn et al. 2001	1	2000	Netherlands	Plant	Grassland	I	44	0.414	0.091
Kleijn et al. 2004	2	2000	Netherlands	Bees	Grassland	I	78	0.867	0.182
Kleijn et al. 2004	2	2000	Netherlands	Bees	Grassland	I	78	0.740	0.125
Kleijn et al. 2004	2	2000	Netherlands	Bees	Grassland	I	78	0.957	0.200
Kleijn et al. 2004	2	2000	Netherlands	Hoverflies	Grassland	I	78	0.378	0.182
Kleijn et al. 2004	2	2000	Netherlands	Hoverflies	Grassland	I	78	0.697	0.125
Kleijn et al. 2004	2	2000	Netherlands	Hoverflies	Grassland	I	78	0.361	0.200
Kleijn et al. 2006*	1	2003	United Kingdom	Bee	Cropland	I	14	0.767	0.286
Kleijn et al. 2006*	1	2003	United Kingdom	Bee	Cropland	I	14	0.632	0.286
Kleijn et al. 2006*	1	2003	United Kingdom	Bee	Cropland	I	14	-0.447	0.286
Kleijn et al. 2006*	1	2003	United Kingdom	Bird	Cropland	I	14	-0.237	0.286
Kleijn et al. 2006*	1	2003	United Kingdom	Bird	Cropland	I	14	0.393	0.286
Kleijn et al. 2006*	1	2003	United Kingdom	Bird	Cropland	I	14	1.066	0.286
Kleijn et al. 2006*	1	2003	United Kingdom	Plant	Cropland	I	14	1.001	0.286
Kleijn et al. 2006*	1	2003	United Kingdom	Plant	Cropland	I	14	0.283	0.286
Kleijn et al. 2006*	1	2003	United Kingdom	Plant	Cropland	I	14	1.140	0.286
Kleijn et al. 2006*	1	2003	United Kingdom	Spider	Cropland	I	14	0.062	0.286
Kleijn et al. 2006*	1	2003	United Kingdom	Spider	Cropland	I	14	0.000	0.286

Appendix S3. Continued.

Publication	*Source*	*Study year*	*Country*	*Organism*	*Habitat*	*AES*	*N*	*g*	*np var g*
Kleijn et al. 2006*	1	2003	United Kingdom	Spider	Cropland	I	14	0.535	0.286
Kleijn et al. 2006*	1	2003	Germany	Bird	Cropland	I	12	0.180	0.333
Kleijn et al. 2006*	1	2003	Germany	Bird	Cropland	I	12	0.390	0.333
Kleijn et al. 2006*	1	2003	Germany	Bird	Cropland	I	14	-0.242	0.286
Kleijn et al. 2006*	1	2003	Germany	Grasshopper	Cropland	I	12	0.458	0.333
Kleijn et al. 2006*	1	2003	Germany	Grasshopper	Cropland	I	12	0.000	0.333
Kleijn et al. 2006*	1	2003	Germany	Grasshopper	Cropland	I	14	0.123	0.286
Kleijn et al. 2006*	1	2003	Netherlands	Bee	Grassland	I	14	-0.578	0.286
Kleijn et al. 2006*	1	2003	Netherlands	Bee	Grassland	I	14	0.141	0.286
Kleijn et al. 2006*	1	2003	Netherlands	Bee	Grassland	I	12	0.000	0.333
Kleijn et al. 2006*	1	2003	Netherlands	Bird	Grassland	I	14	-0.345	0.286
Kleijn et al. 2006*	1	2003	Netherlands	Bird	Grassland	I	14	0.703	0.286
Kleijn et al. 2006*	1	2003	Netherlands	Bird	Grassland	I	12	0.000	0.333
Kleijn et al. 2006*	1	2003	Netherlands	Grasshopper	Grassland	I	14	-0.475	0.286
Kleijn et al. 2006*	1	2003	Netherlands	Grasshopper	Grassland	I	14	0.000	0.286
Kleijn et al. 2006*	1	2003	Netherlands	Grasshopper	Grassland	I	12	-0.331	0.333
Kleijn et al. 2006*	1	2003	Netherlands	Plant	Grassland	I	14	0.368	0.286
Kleijn et al. 2006*	1	2003	Netherlands	Plant	Grassland	I	14	0.429	0.286
Kleijn et al. 2006*	1	2003	Netherlands	Plant	Grassland	I	12	0.132	0.333
Kleijn et al. 2006*	1	2003	Netherlands	Spider	Grassland	I	12	0.285	0.333
Kleijn et al. 2006*	1	2003	Netherlands	Spider	Grassland	I	8	-0.232	0.500
Kleijn et al. 2006*	1	2003	Spain	Bird	Cropland	I	14	0.564	0.286
Kleijn et al. 2006*	1	2003	Spain	Bird	Cropland	I	14	0.566	0.286
Kleijn et al. 2006*	1	2003	Spain	Bird	Cropland	I	14	0.644	0.286
Kleijn et al. 2006*	1	2003	Spain	Grasshopper	Cropland	I	6	0.693	0.667
Kleijn et al. 2006*	1	2003	Spain	Grasshopper	Cropland	I	14	0.000	0.286
Kleijn et al. 2006*	1	2003	Spain	Grasshopper	Cropland	I	14	-0.323	0.286
Kleijn et al. 2006*	1	2003	Spain	Plant	Cropland	I	6	2.339	0.667
Kleijn et al. 2006*	1	2003	Spain	Plant	Cropland	I	14	1.172	0.286
Kleijn et al. 2006*	1	2003	Spain	Plant	Cropland	I	12	0.600	0.333
Kleijn et al. 2006*	1	2003	Spain	Spider	Cropland	I	6	0.753	0.667
Kleijn et al. 2006*	1	2003	Spain	Spider	Cropland	I	14	1.773	0.286
Kleijn et al. 2006*	1	2003	Spain	Spider	Cropland	I	14	0.237	0.286
Kleijn et al. 2006*	1	2003	Switzerland	Bird	Grassland	I	14	0.313	0.286
Kleijn et al. 2006*	1	2003	Switzerland	Bird	Grassland	I	14	0.517	0.286
Kleijn et al. 2006*	1	2003	Switzerland	Bird	Grassland	I	14	0.173	0.286
Knop et al. 2006*	1	2003	Switzerland	Bee	Grassland	I	14	0.560	0.286
Knop et al. 2006*	1	2003	Switzerland	Bee	Grassland	I	14	0.985	0.286
Knop et al. 2006*	1	2003	Switzerland	Bee	Grassland	I	14	0.650	0.286
Knop et al. 2006*	1	2003	Switzerland	Grasshopper	Grassland	I	14	1.804	0.286
Knop et al. 2006*	1	2003	Switzerland	Grasshopper	Grassland	I	14	0.664	0.286
Knop et al. 2006*	1	2003	Switzerland	Grasshopper	Grassland	I	14	0.000	0.286
Knop et al. 2006*	1	2003	Switzerland	Plant	Grassland	I	14	0.408	0.286
Knop et al. 2006*	1	2003	Switzerland	Plant	Grassland	I	14	1.053	0.286
Knop et al. 2006*	1	2003	Switzerland	Plant	Grassland	I	14	2.158	0.286
Knop et al. 2006*	1	2003	Switzerland	Spider	Grassland	I	14	-0.148	0.286
Knop et al. 2006*	1	2003	Switzerland	Spider	Grassland	I	14	0.280	0.286
Knop et al. 2006*	1	2003	Switzerland	Spider	Grassland	I	14	0.406	0.286
Kohler et al. 2008*	2	2005	Netherlands	Bees	Grassland	O	16	1.411	0.220

Appendix S3. Continued.

Publication	*Source*	*Study year*	*Country*	*Organism*	*Habitat*	*AES*	*N*	*g*	*np var g*
Kohler et al. 2008*	2	2005	Netherlands	Hoverflies	Grassland	O	16	2.071	0.220
Kovács-Hostyánszki et al. 2011	2	2008	Hungary	Bees	Cropland	O	33	-1.796	0.229
Kovács-Hostyánszki et al. 2011	2	2008	Hungary	Butterflies	Cropland	O	33	3.547	0.229
Krauss et al. 2011	3	2008	Germany	Plants	Cereal	I	30	4.291	0.133
Krauss et al. 2011	2	2008	Germany	Bumblebees	Cropland	I	30	2.624	0.133
Krauss et al. 2011	2	2008	Germany	Butterflies	Cropland	I	30	1.117	0.133
Krauss et al. 2011	2	2008	Germany	Hoverflies	Cropland	I	30	2.241	0.133
Kruess & Tscharntke 2002a	1	1996	Germany	Auchenorrhyncha	Grassland	I	12	0.673	0.333
Kruess & Tscharntke 2002a	1	1996	Germany	Coleoptera	Grassland	I	12	2.292	0.333
Kruess & Tscharntke 2002a	1	1996	Germany	Heteroptera	Grassland	I	12	1.186	0.333
Kruess & Tscharntke 2002a	1	1996	Germany	Hymenoptera Parasitica	Grassland	I	12	1.349	0.333
Kruess & Tscharntke 2002b	1	1996	Germany	Caelifera	Grassland	I	12	1.348	0.333
Kruess & Tscharntke 2002b	1	1996	Germany	Ensifera	Grassland	I	12	0.873	0.333
Kruess & Tscharntke 2002b	1	1996	Germany	Plant	Grassland	I	12	0.184	0.333
Kruess & Tscharntke 2002b	1	1996	Germany	Trap nesting bee	Grassland	I	12	0.603	0.333
Kruess & Tscharntke 2002b	2	1996	Germany	Butterflies and Burnet moths	Grassland	I	12	1.473	0.333
Kvarnbäck 2009	2	2008	Sweden	Bumblebees	Cropland	O	12	1.046	0.400
Kvarnbäck 2009	2	2008	Sweden	Butterflies	Cropland	O	12	1.225	0.400
Macfadyen et al. 2009	3	2006	United Kingdom	Arthropods	Cereal	I	20	1.237	0.200
Macfadyen et al. 2009	3	2006	United Kingdom	Arthropods	Cereal	I	20	1.031	0.200
Macfadyen et al. 2009	3	2006	United Kingdom	Plants	Cereal	I	20	1.084	0.200
Mand et al. 2001	2	2000	Estonia	Bumblebees	Cropland and grassland	I	24	0.821	0.167
Manhoudt et al. 2007	1	2003	Netherlands	Plant	Grassland	I	10	1.984	0.417
Manhoudt et al. 2007	1	2003	Netherlands	Plant	Grassland	I	28	1.084	0.146
Marshall et al. 2006*	1	2003	United Kingdom	Grasshopper	Grassland	I	14	0.323	0.286
Marshall et al. 2006*	1	2003	United Kingdom	Grasshopper	Grassland	I	14	1.332	0.286
Marshall et al. 2006*	1	2003	United Kingdom	Grasshopper	Grassland	I	14	1.138	0.286
Meek et al. 2002	2	1999	United Kingdom	Butterflies	Cropland	O	8	-0.037	0.500
Meek et al. 2002	2	1999	United Kingdom	Butterflies	Cropland	O	8	0.971	0.500
Merckx et al. 2009	2	2006	United Kingdom	Larger moths	Cropland	O	48	1.369	0.500
Merckx et al. 2009	2	2006	United Kingdom	Larger moths	Cropland	O	48	1.553	0.500
Moreby et al. 1994	1	1991	United Kingdom	Plant	Cropland	I	62	3.160	0.065
Muchow et al. 2007	2	2006	Germany	Bees	Cropland	O	45	1.995	0.278
Muchow et al. 2007	2	2006	Germany	Butterflies	Cropland	O	18	2.054	0.278
Nickel & Achtziger 2005	1	1996	Germany	Leafhoppers	Grassland	I	8	0.962	0.667
Nickel & Achtziger 2005	1	1996	Germany	Leafhoppers	Grassland	I	9	2.198	0.643
Öberg 2007	1	2004	Sweden	Linyphiidae	Cropland	I	8	-1.541	0.533
Peter & Walter 2001	1	2000	Switzerland	Grasshopper	Grassland	I	304	0.301	0.013
Petersen et al. 2006	1	2002	Denmark	Plant	Grassland	I	40	1.352	0.100
Ponce et al. 2011	3	2008	Spain	Arthropods	Cereal	I	56	0.578	0.071
Ponce et al. 2011	3	2008	Spain	Plants	Cereal	I	56	1.907	0.071
Power & Stout 2011	3	2009	Ireland	Plants	Grass	I	20	1.007	0.200
Power & Stout 2011	2	2009	Ireland	Bees	Grassland	I	20	0.609	0.200
Power & Stout 2011	2	2009	Ireland	Hoverflies	Grassland	I	20	0.127	0.200
Purtauf et al. 2005	3	2002	Germany	Arthropods	Cereal	I	24	-0.269	0.167
Pywell et al. 2005	2	2003	United Kingdom	Bumblebees	Cropland	O	76	2.725	0.125
Pywell et al. 2005	2	2003	United Kingdom	Bumblebees	Cropland	O	86	2.334	0.071
Pywell et al. 2006	2	2004	United Kingdom	Bumblebees	Cropland	O	64	1.363	0.063
Pywell et al. 2006	2	2004	United Kingdom	Bumblebees	Cropland	O	64	2.888	0.075

Appendix S3. Continued.

Publication	*Source*	*Study year*	*Country*	*Organism*	*Habitat*	*AES*	*N*	*g*	*np var g*
Reddersen 1997	3	1988	Denmark	Arthropods	Cereal	I	34	3.462	0.118
Risberg 2004	2	2002	Sweden	Bumblebees	Cropland	I	10	0.140	0.400
Romero et al. 2008	1	2004	Spain	Plant	Cropland	I	36	2.044	0.111
Roschewitz et al. 2005	1	2002	Germany	Plant	Cropland	I	24	2.310	0.167
Roth et al. 2008	2	2000	Switzerland	Butterflies	Cropland and grassland	I	87	0.448	0.048
Rundlöf & Smith 2006	2	2004	Sweden	Butterflies and Burnet moths	Cropland	I	24	0.561	0.333
Rundlöf & Smith 2006	2	2004	Sweden	Butterflies and Burnet moths	Cropland	I	24	1.890	0.333
Rundlöf et al. 2010	3	2004	Sweden	Plants	Mixed	I	14	2.761	0.286
Rundlöf et al. 2010	3	2004	Sweden	Plants	Mixed	I	14	2.196	0.286
Rundlöf et al. 2010	3	2004	Sweden	Plants	Mixed	I	14	2.927	0.286
Rundlöf et al. 2008a	2	2005	Sweden	Butterflies and Burnet moths	Cropland	I	16	3.867	0.250
Rundlöf et al. 2008b	2	2004	Sweden	Bumblebees	Cropland	I	24	0.648	0.333
Rundlöf et al. 2008b	2	2004	Sweden	Bumblebees	Cropland	I	24	2.289	0.333
Salonen & Hyvönen 2011	3	1999	Finland	Plants	Cereal	I	595	1.922	0.016
Salonen et al. 2001	3	1999	Finland	Plants	Cereal	I	30	0.272	0.133
Schmidt et al. 2005	1	2002	Germany	Spider	Cropland	I	24	-0.143	0.167
Sepp et al. 2005	1	2002	Estonia	EArthropodsworm	Cropland	I	15	0.074	0.300
Sepp et al. 2005	1	2002	Estonia	EArthropodsworm	Cropland	I	15	0.073	0.300
Shah et al. 2005	1	1994	United Kingdom	Carabid	Cropland	I	20	-0.771	0.200
Shah et al. 2005	1	1994	United Kingdom	Rove beetle	Cropland	I	20	0.000	0.200
Sjödin et al. 2008	2	2004	Sweden	Bees	Grassland	I	16	0.081	0.250
Sjödin et al. 2008	2	2004	Sweden	Butterflies and Burnet moths	Grassland	I	16	-0.111	0.250
Sjödin et al. 2008	2	2004	Sweden	Hoverflies	Grassland	I	16	0.652	0.250
Smith et al. 2010	3	2005	Sweden	Birds	Cereal	I	24	1.032	0.167
Smith et al. 2010	3	2005	Sweden	Birds	Cereal	I	24	-0.153	0.167
Steffan-Dewenter & Tscharntke 1997	2	1992	Germany	Butterflies	Cropland	O	8	5.942	0.500
Steffan-Dewenter & Tscharntke 1997	2	1992	Germany	Butterflies	Cropland	O	8	2.341	0.500
Steffan-Dewenter & Tscharntke 2001	2	1993	Germany	Bees	Cropland	O	8	1.425	0.500
Steffan-Dewenter & Tscharntke 2001	2	1993	Germany	Bees	Cropland	O	8	-0.464	0.500
Ulber et al. 2009	3	2007	Germany	Plants	Cereal	I	16	0.850	0.250
Van der Gast et al. 2011	3	2003	United Kingdom	Microbes	Mixed	I	18	0.549	0.222
van Diepingen et al. 2006	3	2001	Netherlands	Microbes	Mixed	I	28	0.854	0.143
van Diepingen et al. 2006	3	2001	Netherlands	Nematodes	Mixed	I	28	0.572	0.143
Verbruggen et al. 2010	3	2007	Netherlands	Microbes	Cereal	I	26	0.844	0.154
Weibull & Östman 2003	3	1998	Sweden	Arthropods	Mixed	I	16	-0.075	0.250
Weibull & Östman 2003	3	1998	Sweden	Arthropods	Mixed	I	16	0.962	0.250
Weibull & Östman 2003	3	1999	Sweden	Arthropods	Mixed	I	16	-0.027	0.250
Weibull & Östman 2003	3	1999	Sweden	Arthropods	Mixed	I	16	-0.278	0.250
Weibull & Östman 2003	3	1999	Sweden	Arthropods	Mixed	I	16	-1.058	0.250
Weibull & Östman 2003	3	1999	Sweden	Arthropods	Mixed	I	16	-0.096	0.250
Weibull & Östman 2003	3	1999	Sweden	Arthropods	Mixed	I	16	-0.611	0.250
Weibull & Östman 2003	3	1999	Sweden	Arthropods	Mixed	I	16	-0.817	0.250
Weibull & Östman 2003	3	1999	Sweden	Arthropods	Mixed	I	16	-0.369	0.250
Weibull & Östman 2003	3	1999	Sweden	Arthropods	Mixed	I	16	-0.062	0.250
Weibull & Östman 2003	3	1998	Sweden	Plants	Mixed	I	16	0.301	0.250
Weibull & Östman 2003	3	1998	Sweden	Plants	Mixed	I	16	-0.190	0.250
Winqvist et al. 2011	3	2007	Sweden, Estonia, W+E Germany, Netherlands	Arthropods	Cereal	I	151	0.065	0.030
Winqvist et al. 2011	3	2007	Sweden, Estonia, W+E Germany, Netherlands	Birds	Cereal	I	151	0.227	0.030
Winqvist et al. 2011	3	2007	Sweden, Estonia, W+E Germany, Netherlands	Plants	Cereal	I	151	0.451	0.030
Yeats et al. 1997	3	1994	United Kingdom	Protozoa	Grass	I	6	0.285	0.667

Literature Cited

Aavik, T. and Liira, J. 2010. Quantifying the effect of organic farming, field boundary type and landscape structure on the vegetation of field boundaries. Agriculture, Ecosystems and Environment **135**:178–186.

Albrecht, M., Duelli, P., Müller, C., Kleijn, D. and Schmid, B. 2007a. The Swiss agri-environment scheme enhances pollinator diversity and plant reproductive success in nearby intensively managed farmland. Journal of Applied Ecology **44**:813-822.

Albrecht, M., Duelli, P., Schmid, B. and Müller, C.B. 2007b. Interaction diversity within quantified insect food webs in restored and adjacent intensively managed meadows. Journal of Animal Ecology **76**:1015–1025.

Albrecht, M., Schmid, B., Obrist, M.K., Schüpbach, B., Kleijn, D. and Duelli, P. 2010. Effects of ecological compensation meadows on arthropod diversity in adjacent intensively managed grassland. Biological Conservation **143**:642–649.

Alvarez, T., Frampton, G.K. and Goulson, D. 2001. Epigeic Collembola in winter wheat under organic, integrated and conventional farm management regimes. Agriculture, Ecosystems and Environment **83**:95–110.

Aude, E., Tybirk, K. and Pedersen, M.B. 2003. Vegetation diversity of conventional and organic hedgerows in Denmark. Agriculture Ecosystems and Environment **99**:135–147.

Aviron, S., Nitsch, H., Jeanneret, P., Buholzer, S., Luka, H., Pfiffner, L. et al. 2009. Ecological cross compliance promotes farmland biodiversity in Switzerland. Frontiers in Ecology and the Environment **7**:247–252.

Aviron, S., Herzog, F., Klaus, I., Schüpbach, B. and Jeanneret, P. 2010. Effects of wildflower strip quality, quantity, and connectivity on butterfly diversity in a Swiss arable landscape. Restoration Ecology **19**:500–508.

Batáry, P., Matthiesen, T. and Tscharntke, T. 2010. Landscape-moderated importance of hedges in conserving farmland bird diversity of organic vs. conventional croplands and grasslands. Biological Conservation **143**:2020–2027.

Batáry, P., Holzschuh, A., Orci, K.M., Samu, F. and Tscharntke, T. 2012. Responses of plant, insect and spider biodiversity to local and landscape scale management intensity in cereal crops and grasslands. Agriculture, Ecosystems and Environment **146**:130–136

Brittain, C., Bommarco, R., Vighi, M., Settele, J. and Potts, S.G. 2010. Organic farming in isolated landscapes does not benefit flower-visiting insects and pollination. Biological Conservation **143**:1860–1867.

Bruggisser, O.T., Schmidt-Entling, M.H. and Bacher, S. 2010. Effects of vineyard management on biodiversity at three trophic levels. Biological Conservation **143**:1521–1528.

BTO. 1995. The Effect of Organic Farming Regimes on Breeding and Winter Bird Populations. Research report No.154. British Trust for Ornithology: Norfolk, UK.

Caballero-Lopez, B., Blanco-Moreno, J.M., Perez, N., Pujade-Villar, J., Ventura, D., Oliva, F. and Sans, F.X. 2010. A functional approach to assessing plant-arthropod interaction in winter wheat. Agriculture, Ecosystems and Environment **137**:288–293.

Carvell, C., Meek, W.R., Pywell, R.F., Goulson, D. and Nowakowski, M. 2007. Comparing the efficacy of agri-environment schemes to enhance bumble bee abundance and diversity on arable field margins. Journal of Applied Ecology **44**:29–40.

Christensen, K.D., Jacobsen, E.M. and Nøhr, H. 1996. A comparative study of bird faunas in conventionally and organically farmed areas. Dansk Ornithologisk Forenings Tidskrift **90**:21–28.

Clough, Y., Holzschuh, A., Gabriel, D., Purtauf, T., Kleijn, D., Kruess, A., Steffan-Dewenter, I. and Tscharntke, T. 2007a. Alpha and beta diversity of arthropods and plants in organically and conventionally managed wheat fields. Journal of Applied Ecology **44**:804–812

Clough, Y., Kruess, A. and Tscharntke, T. 2007b. Organic versus conventional arable farming systems: Functional grouping helps understand staphylinid response. Agriculture, Ecosystems and Environment **118**:285–290.

Concepción, E.D., Díaz, M. and Baquero, R.A. 2008. Effects of landscape complexity on the ecological effectiveness of agri-environment schemes. Landscape Ecology **23**:135–148.

Dänhardt, J., Green, M., Lindström, Å., Rundlöf, M. and Smith, H.G. 2010. Farmland as stopover habitat for migrating birds – effects of organic farming and landscape structure. Oikos, 119, 1114–1125.

de Snoo, G.R., Van der Poll, R.J. and Bertels, J. 1998. Butterflies in sprayed and unsprayed field margins. Journal of Applied Entomology **122**:157–161.

Diekötter, T., Wamser, S., Wolters, V. and Birkhofer, K. 2010. Landscape and management effects on structure and function of soil arthropod communities in winter wheat. Agriculture, Ecosystems and Environment **137**:108–112.

Döring, T.F., Hiller, A., Wehke, S., Schulte, G. and Broll, G. 2003. Biotic indicators of carabid species richness on organically and conventionally managed arable fields. Agriculture, Ecosystems and Environment **98**:133–139.

Ekroos, J., Piha, M. and Tiainen, J. 2008. Role of organic and conventional field boundaries on boreal bumblebees and butterflies. Agriculture, Ecosystems and Environment **124**:155–159.

Ekroos, J., Heliölä, J. and Kuussaari, M. 2010. Homogenization of lepidopteran communities in intensively cultivated agricultural landscapes. Journal of Applied Ecology **47**:459–467.

Feber, R.E., Bell, J., Johnson, P.J., Firbank, L.G. and Macdonald, D.W. 1998. The effects of organic farming on surface-active spider Araneae assemblages in wheat in Southern England, UK. Journal of Arachnology **26**:190–202.

Feber, R.E., Johnson, P.J., Firbank, L.G., Hopkins, A. and Macdonald, D.W. 2007. A comparison of butterfly populations on organically and conventionally managed farmland. Journal of Zoology **273**:30–39.

Fischer, C., Flohre, A., Clement, L.W., Batáry, P., Weisser, W.W., Tscharntke, T. and Thies, C. 2011a. Mixed effects of landscape structure and farming practice on bird diversity. Agriculture, Ecosystems and Environment **141**:119–125.

Fischer, C., Thies, C. and Tscharntke, T. 2011b. Small mammals in agricultural landscapes: Opposing responses to farming practices and landscape complexity. Biological Conservation **144**:1130–1136.

Flohre, A., Rudnick, M., Traser, G., Tscharntke, T. and Eggers, T. 2011. Does soil biota benefit from organic farming in complex vs. simple landscapes? Agriculture, Ecosystems and Environment **141**:210–214.

Fuentes-Montemayor, E., Goulson, D. and Park, K.J. 2011. The effectiveness of agri-environment schemes for the conservation of farmland moths: assessing the importance of a landscape-scale management approach. Journal of Applied Ecology **48**:532–542.

Gabriel, D., Roschewitz, I., Tscharntke, T. and Thies, C. 2006. Beta diversity at different spatial scales: plant communities in organic and conventional agriculture. Ecological Applications **16**:2011–2021.

Gabriel, D., Sait, S.M., Hodgson, J.A., Schmutz, U., Kunin, W.E. and Benton, T.G. 2010. Scale matters: the impact of organic farming on biodiversity at different spatial scales. Ecology Letters **13**:858–869.

Galván, G.A., Parádi, I., Burger, K., Baar, J., Kuyper, T.W., Scholten, O.E. and Kik, C. 2009. Molecular diversity of arbuscular mycorrhizal fungi in onion roots from organic and conventional farming systems in the Netherlands. Mycorrhiza **19**:317–328.

Gathmann, A., Greiler, H.J. and Tscharntke, T. 1994. Trap-nesting bees and wasps colonizing set-aside fields: succession and body size, management by cutting and sowing. Oecologia **98**:8–14.

Genghini, M., Gellini, S. and Gustin, M. 2006. Organic and integrated agriculture: the effects on bird communities in orchard farms in northern Italy. Biodiversity and Conservation **15**:3077–3094.

Granqvist, Å. 1999. Mångfalden Av Växter i Jordbrukslandskapet – Effekten Av Odlingssystem Och Landskapsheterogenitet. Examensarbeten/Seminarieuppsatser 4. Diversity of Plants in the Agricultural Landscape. BSc Report. Department of Ecology and Crop Production Science, SLU. Uppsala.

Haenke, S., Scheid, B., Schaefer, M., Tscharntke, T. and Thies, C. 2009. Increasing syrphid fly diversity and density in sown flower strips within simple vs. complex landscapes. Journal of Applied Ecology **46**:1106–1114.

Hawes, C., Squire, G.R., Hallett, P.D., Watson, C.A. and Young, M. 2010. Arable plant communities as indicators of farming practice. Agriculture, Ecosystems and Environment **138**:17–26.

Hodgson, J.A., Kunin, W.E., Thomas, C.D., Benton, T.G. and Gabriel, D. 2010. Comparing organic farming and land sparing: optimizing yield and butterfly populations at a landscape scale. Ecol. Lett., 13, 1358–1367.

Hokkanen, H. and Holopainen, J.K. 1986. Carabid species and activity densities in biologically and conventionally managed cabbage fields. Journal of Applied Entomology **102**:353–363.

Holzschuh, A., Steffan-Dewenter, I., Kleijn, D. and Tscharntke, T. 2007. Diversity of flower-visiting bees in cereal fields: effects of farming system, landscape composition and regional context. Journal of Applied Ecology **44**:41–49.

Hutton, S.A. and Giller, P.S. 2003. The effects of the intensification of agriculture on northern temperate dung beetle communities. Journal of Applied Ecology **40**:994–1007.

Hyvonen, T., Ketoja, E., Salonen, J., Jalli, H. and Tiainen, J. 2003 Weed species diversity and community composition in organic and conventional cropping of spring cereals. Agriculture, Ecosystems and Environment **97**:131–149.

Irmler, U. 2003. The spatial and temporal pattern of carabid beetles on arable fields in northern Germany Schleswig-Holstein and their value as ecological indicators. Agriculture, Ecosystems and Environment **98**:141–151.

Jonason, D., Andersson, G.K.S., Öckinger, E., Rundlöf, M., Smith, H.G. and Bengtsson, J. 2011. Assessing the effect of the time since transition to organic farming on plants and butterflies. Journal of Applied Ecology **48**:543–550.

Jose-Maria L.; Sans F. X. 2011. Weed seedbanks in arable fields: effects of management practices and surrounding landscape. Weed Research **51**:631–640.

Kleijn, D., Boekhoff, M. and Ottburg, F. 1999. Een studie naar de effectiviteit van beheersovereenkomsten in de polders Westbroek en Maarsseveen. Wageningen UR, Wageningen.

Kleijn, D., Berendse, F., Smit, R., Gilissen, N., Smit, J., Brak, B. et al. 2004. Ecological effectiveness of agri-environment schemes in different agricultural landscapes in the Netherlands. Conservation Biology **18**:775–786.

Kleijn, D., Baquero, R.A., Clough, Y., Díaz, M., De Esteban, J., Fernández, F., Gabriel, D., Herzog, F., Holzschuh, A., Jöhl, R., Knop, E., Kruess, A., Marshall, E.J.P., Steffan-Dewenter, I., Tscharntke, T., Verhulst, J., West, T.M. and Yela, J.L. 2006. Mixed biodiversity benefits of agri-environment schemes in five European countries. Ecology Letters **9**:243–254.

Kleijn, D., Berendse, F., Smit, R. and Gilissen, N. 2001. Agri-environment schemes do not effectively protect biodiversity in Dutch agricultural landscapes. Nature **413**:723–725.

Knop, E., Kleijn, D., Herzog, F. and Schmid, B. 2006. Effectiveness of the Swiss agri-environment scheme in promoting biodiversity. Journal of Applied Ecology **43**:120–127.

Kohler, F., Verhulst, J., Van Klink, R. and Kleijn, D. 2008. At what spatial scale do high-quality habitats enhance the diversity of forbs and pollinators in intensively farmed landscapes? Journal of Applied Ecology **45**:753–762.

Kovács-Hostyánszki, A., Kőrösi, A., Orci, K.M. Batáry, P. and Báldi, A. 2011. Set-aside promotes insect and plant diversity in a Central European country. Agriculture, Ecosystems and Environment **141**:296–301.

Kruess, A., and Tscharntke, T. 2002a. Contrasting responses of plant and insect diversity to variation in grazing intensity. Biological Conservation **106**:293–302.

Kruess, A., and Tscharntke, T. 2002b. Grazing intensity and the diversity of Orthoptera, butterflies and trapnesting bees and wasps. Conservation Biology **16**:1570–1580.

Kvarnbäck, O. 2009. Förbättrad överlevnad av fågelungar på ekologiska fält: försök med lärkrutor och kantzoner. Hushållningssällskapet.

Macfadyen, S., Gibson, R., Polaszek, A., Morris, R.J., Craze, P.G., Planque, R., Symondson, W.O.C. and Memmott, J. 2009. Do differences in food web structure between organic and conventional farms affect the ecosystem service of pest control? Ecology Letters **12**:229–238.

Mänd, M., Geherman, V., Luik, A., Martin, A., Mikk, M., Paimetova, V. et al. 2001. Bumblebee diversity on ecological and conventional dairy farms. Acta Biologica Universitutis Dauguvpiliereslis **1**:21–25.

Manhoudt, A.G.E., Visser, A.J. and de Snoo, G.R. 2007. Management regimes and farming practices enhancing plant species richness on ditch banks. Agriculture, Ecosystems and Environment **119**:353–358.

Marshall, E.J.P., West, T.M. and Kleijn, D. 2006. Impacts of an agri-environment field margin prescription on the flora and fauna of arable farmland in different landscapes. Agriculture, Ecosystems and Environment **113**:36–47.

Meek, B., Loxton, D., Sparks, T., Pywell, R., Pickett, H. and Nowakowski, M. 2002. The effect of arable field margin composition on invertebrate Biodiversity. Biological Conservation **106**:259–271.

Merckx, T, Feber, R.E., Riordan, P., Townsend, M.C., Bourn, N.A.D., Parsons, M.S. et al. 2009. Optimizing the biodiversity gain from agri-environment schemes. Agriculture, Ecosystems and Environment **130**:177–182.

Moreby, S.J., Aebischer, N.J., Southway, S.E. and Sotherton, N.W. 1994. A comparison of the flora and arthropod fauna of organically and conventionally grown winter wheat in Southern England. Annals of Applied Biology **125**:13–27.

Muchow, T., Becker, A., Schindler, M. and Wetterich, F. 2007. Naturschutz in Börde-Landschaften durch Strukturelemente am Beispiel der Köner-Bucht. Abschlussbericht des DBV Bördeprojektes. Gefördert durch die Deutsche Bundestiftung Umwelt DBU.

Nickel, H. and Achtziger, R. 2005. Do they ever come back? Responses of leafhopper communities to extensification of land use. Journal of Insect Conservation **9**:319–333.

Öberg, S. 2007. Diversity of spiders after spring sowing – influence of farming system and habitat type. Journal of Applied Entomology **131**:524–531.

Peter, B. and Walter, T. 2001. Grasshopers need ecological compensation areas. Agrarforschung **8**:452–457.

Petersen, S., Axelsen, J.A., Tybirk, K., Aude, E. and Vestergaard, P. 2006. Effects of organic farming on field boundary vegetation in Denmark. Agriculture, Ecosystems and Environment **113**:302–306.

Ponce, C., Bravo, C., Garcia De Leon, D., Magana, M. and Alonso, J.C. 2011. Effects of organic farming on plant and arthropod communities: A case study in Mediterranean dryland cereal. Agriculture, Ecosystems and Environment **141**:193–201.

Power, E.F. and Stout, J.C. 2011. Organic dairy farming: impacts on insect–flower interaction networks and pollination. Journal of Applied Ecology **48**:561–569.

Purtauf, T., Roschewitz, I., Dauber, J., Thies, C., Tscharntke, T. and Wolters, V. 2005 Landscape context of organic and conventional farms: Influences on carabid beetle diversity. Agriculture, Ecosystems and Environment **108**:165–174.

Pywell, R.F., Warman, E.A., Carvell, C., Sparks, T.H., Dicks, L.V., Bennett, D. et al. 2005. Providing foraging resources for bumblebees in intensively farmed landscapes. Biological Conservation **121**:479–494.

Pywell, R.F., Warman, E.A., Hulmes, L., Hulmes, S., Nuttall, P., Sparks, T.H. et al. 2006. Effectiveness of new agri-environment schemes in providing foraging resources for bumblebees in intensively farmed landscapes. Biological Conservation **129**:192–206.

Reddersen, J. 1997. The Arthropod Fauna of Organic Versus Conventional Cereal Fields in Denmark. Biological Agriculture and Horticulture **15**:61–71.

Risberg, J.O. 2004. Humlor Bombus på ekologiska och konventionella gårdar: odlingssystemets och landskapets betydelse för en ekologisk nyckelresurs. Svenska Vildbiprojektet vid ArtDatabanken, SLU och Avdelningen för Växtekologi, Uppsala Universitet.

Romero, A., Chamorro, L. and Sans, F.X. 2008. Weed diversity in crop edges and inner fields of organic and conventional dryland winter cereal crops in NE Spain. Agriculture, Ecosystems and Environment **124**:97–104.

Roschewitz, I., Gabriel, D., Tscharntke, T. and Thies, C. 2005. The effects of landscape complexity on arable weed species diversity in organic and conventional farming. Journal of Applied Ecology **42**:873–882.

Roth, T., Amrhein, V., Peter, B. and Weber, D. 2008. A Swiss agri-environment scheme effectively enhances species richness for some taxa over time. Agriculture, Ecosystems and Environment **125**:167–172.

Rundlöf, M. and Smith, H.G. 2006. The effect of organic farming on butterfly diversity depends on landscape context. Journal of Applied Ecology **43**:1121–1127.

Rundlöf, M., Bengtsson, J. and Smith, H.G. 2008a. Local and landscape effects of organic farming on butterfly species richness and abundance. Journal of Applied Ecology **45**:813–820.

Rundlöf, M., Nilsson, H. and Smith, H.G. 2008b. Interacting effects of farming practice and landscape context on bumble bees. Biological Conservation **141**:417–426.

Rundlöf, M., Edlund, M. and Smith, H.G. 2010 Organic farming at local and landscape scales benefits plant diversity. Ecography **33**:514–522.

Salonen, J. and Hyvönen, T. 2011. From surveys to an indicator – a case study with arable weeds. NJF Seminar 442. Nordic Meeting on Agricultural Statistics – A joint workshop of statisticians and research community. NJF: Oslo, Norway.

Salonen, J., Hyvönen, T. and Jalli, H. 2001. Weeds in spring cereal fields in Finland – a third survey. Agricultural and Food Science **10**:347–364.

Schmidt, M.H., Roschewitz, I., Thies, C. and Tscharntke, T. 2005. Differential effects of landscape and management on diversity and density of ground-dwelling farmland spiders. Journal of Applied Ecology **42**:281–287.
Sepp, K., Ivask, M., Kaasik, A., Mikk, M. and Peepson, A. 2005. Soil biota indicators for monitoring the Estonian agri-environmental programme. Agriculture, Ecosystems and Environment **108**:264–273.
Shah, P.A., Brooks, D.R., Ashby, J.E., Perry, J.N. and Woiwod, I.P. 2003. Diversity and abundance of the coleopteran fauna of organic and conventional management systems in Southern England. Agricultural and Forest Entomology **5**:51–60.
Sjödin, N.E., Bengtsson, J. and Ekbom, B. 2008. The influence of grazing intensity and landscape composition on the diversity and abundance of flower-visiting insects. Journal of Applied Ecology **45**:763–772
Sjödin, N.E., Bengtsson, J. and Ekbom, B. 2008. The influence of grazing intensity and landscape composition on the diversity and abundance of flower-visiting insects. Journal of Applied Ecology **45**:763–772.
Smith, H.G., Dänhardt, J., Lindström, Å. and Rundlöf, M. 2010. Consequences of organic farming and landscape heterogeneity for species richness and abundance of farmland birds. Oecologia **162**:1071–1079.
Steffan-Dewenter, I. and Tscharntke, T. 1997. Early succession of butterfly and plant communities on set-aside fields. Oecologia **109**:294–302.
Steffan-Dewenter, I. and Tscharntke, T. 2001. Succession of bee communities on fallows. Ecography **24**:83–93.
Ulber, L., Steinmann, H.H., Klimek, S. and Isselstein, J. 2009. An on-farm approach to investigate the impact of diversified crop rotations on weed species richness and composition in winter wheat. Weed Research **49**:534–543.
van Diepeningen, A.D., de Vos, O.J., Korthals, G.W. and van Bruggen, A.H.C. 2006. Effects of organic versus conventional management on chemical and biological parameters in agricultural soils. Applied Soil Ecology **31**:120–135.
van der Gast, C.J., Gosling, P., Tiwari, B. and Bending, G.D. 2011. Spatial scaling of arbuscular mycorrhizal fungal diversity is affected by farming practice. Environmental Microbiology **13**:241–249.
Verbruggen, E., Röling, W.F.M., Gamper, H.A., Kowalchuk, G.A., Verhoef, H.A. and van der Heijden, M.G.A. 2010. Positive effects of organic farming on below-ground mutualists: large-scale comparison of mycorrhizal fungal communities in agricultural soils. New Phytologist **186**:968–979.
Weibull, A.C. and Östman, O. 2003. Species composition in agroecosystems: The effect of landscape, habitat, and farm management. Basic and Applied Ecology **4**:349–361.
Winqvist, C., Bengtsson, J., Aavik, T., Berendse, F., Clement, L.W., Eggers, S., Fischer, C., Flohre, A., Geiger, F., Liira, J., Pärt, T., Thies, C., Tscharntke, T., Weisser, W.W. and Bommarco, R. 2011. Mixed effects of organic farming and landscape complexity on farmland biodiversity and biological control potential across Europe. Journal of Applied Ecology **48**:570–579.
Yeates, G.W., Bardgett, R.D., Cook, R., Hobbs, P.J., Bowling, P.J. and Potter, J.F. 1997. Faunal and Microbial Diversity in Three Welsh Grassland Soils Under Conventional and Organic Management Regimes. Journal of Applied Ecology **34**:453–470.

Appendix S4. Funnel plot, regression test and fail-safe number.

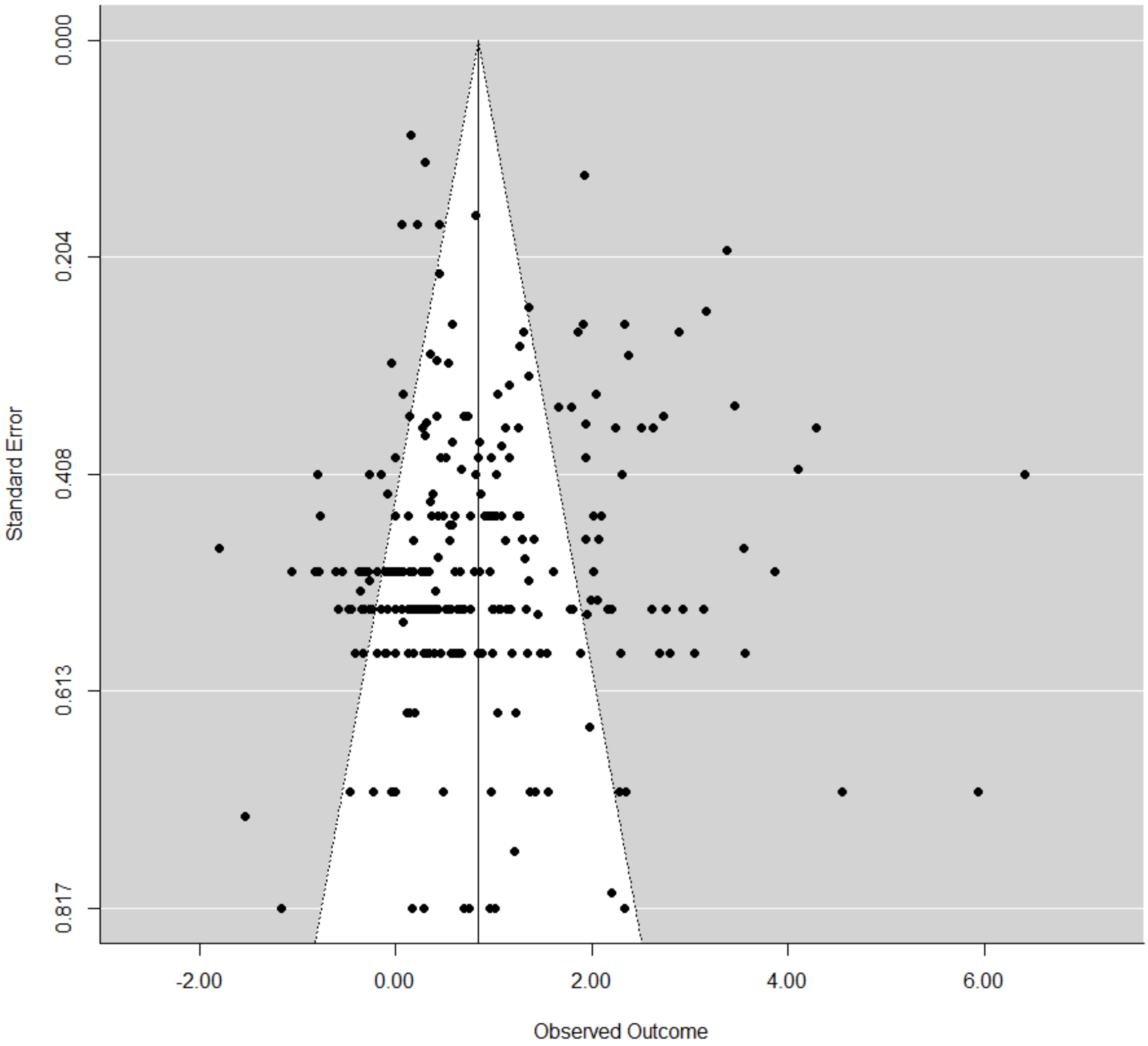

Regression test for funnel plot asymmetry in meta-analysis with the moderator budget period (predictor: sample size): z = 0.834, p = 0.405

Regression test for funnel plot asymmetry in meta-analysis with the moderator AES type (predictor: sample size): z = 0.815, p = 0.415

Rosenthal fail-safe number (target level p = 0.05): 111848

Appendix S5. Summary table of meta-analyses showing tests of moderator and residual heterogeneities and inconsistency indexes.

	d.f.	*Q*	*P*	I^2 *(%)*
Period				
Moderator	1	0.06	0.814	85.6
Residual	282	1975.41	<0.001	
AES type				
Between groups	1	17.20	<0.001	84.8
Within groups	66	1889.49	<0.001	

Appendix S6. ISO2 codes with country names for the 30 countries having AES in the continent. Further country codes are available at URL: https://www.iso.org/obp/ui/#search

Code	Country
AT	Austria
BE	Belgium
BG	Bulgaria
CH	Switzerland
CY	Cyprus
CZ	Czech Republic
DK	Denmark
EE	Estonia
FI	Finland
FR	France
DE	Germany
GR	Greece
HR	Croatia
HU	Hungary
IE	Ireland
IT	Italy
LV	Latvia
LT	Lithuania
LU	Luxembourg
MT	Malta
NL	Netherlands
NO	Norway
PL	Poland
PT	Portugal
RO	Romania
SK	Slovakia
SI	Slovenia
ES	Spain
SE	Sweden
UK	United Kingdom

Appendix S7. Further discussion on "How cost-effective are AES compared to other conservation approaches such as protected farmland areas?"

AES and protected areas do not have to be opposing strategies. With careful spatial planning, they can work together as co-ordinated landscape-scale conservation. For example, AES can be used to create lower-intensity buffers around protected areas to increase their effective size. So far, AES have rarely been targeted in this fashion and the effectiveness of such an approach is entirely untested.

On the other hand, in given cases, AES and protected areas as opposing strategies cannot be separated. For example, in Hungary about 10-20 % of the income of some national park directories with significant areas of semi-natural grasslands comes from AES (István Szentirmai, Őrség National Park Directorate, pers. comm.). This means that AES are used to maintain the protected areas (Báldi et al. 2013).

In principle, AES could also be used to take larger areas of farmland out of production, to protect or restore wild habitats or deliver ecosystem services at catchment scale. Both types of spatial AES planning could be implemented by promoting collaboration between neighbouring farmers. This is already in action in Switzerland (Anonymous 2014), and has been proposed as part of the new AES offer in England (McKenzie et al. 2013).

Literature Cited

Anonymous. 2014. Swiss Confederation. Bundesamt für Landwirtschaft BLW. Ökologie. Available from URL: http://www.blw.admin.ch/dokumentation/01535/01544/index.html?lang=de (accessed October 2014).

Báldi, A., P. Batáry, and D. Kleijn. 2013. Effects of grazing and biogeographic regions on grassland biodiversity in Hungary - analysing assemblages of 1200 species. Agriculture, Ecosystems and Environment **166**:28–34.

McKenzie, A. J., S. B. Emery, J. R. Franks, and M. J. Whittingham. 2013. Landscape-scale conservation: collaborative agri-environment schemes could benefit both biodiversity and ecosystem services, but will farmers be willing to participate? Journal of Applied Ecology **50**:1274–1280.
